# A large explosive silicic eruption in the British Palaeogene Igneous Province

**DOI:** 10.1038/s41598-018-35855-w

**Published:** 2019-01-24

**Authors:** Valentin R. Troll, C. Henry Emeleus, Graeme R. Nicoll, Tobias Mattsson, Robert M. Ellam, Colin H. Donaldson, Chris Harris

**Affiliations:** 1 Department of Earth Sciences, Section for Mineralogy, Petrology, Tectonics (MPT), Uppsala University, Villavägen 16, 752 36 Uppsala, Sweden; 2 Department of Earth Sciences, Durham University, South Road, Durham, DH1 3LE UK; 3 Neftex Insights, Halliburton, 97 Jubilee Avenue, Milton Park, Abingdon, Oxfordshire OX14 4RW UK; 4 Scottish Universities Environmental Research Centre (SUERC), Scottish Enterprise Technology Park, Rankine Avenue, East Kilbride G75 0QF UK; 5 Department of Geography and Geosciences, University of St Andrews, North Street, St Andrews, KY16 9AL UK; 6 Department of Geological Sciences, University of Cape Town, Rondebosch, 7701 South Africa

## Abstract

Large-volume pyroclastic eruptions are not known from the basalt-dominated British Palaeogene Igneous Province (BPIP), although silicic magmatism is documented from intra-caldera successions in central volcanoes and from small-volume ash-layers in the associated lava fields. Exceptions are the Sgùrr of Eigg (58.7 Ma) and Òigh-sgeir pitchstones in the Inner Hebrides (>30 km apart), which have been conjectured to represent remnants of a single large silicic event. Currently available major element data from these outcrops differ, however, creating a need to test if the two pitchstones are really related. We employ a systematic array of methods ranging from mineralogy to isotope geochemistry and find that samples from the two outcrops display identical mineral textures and compositions, major- and trace elements, and Sr-Nd-Pb-O isotope ratios, supporting that the two outcrops represent a single, formerly extensive, pyroclastic deposit. Available isotope constraints suggest a vent in the Hebridean Terrane and available radiometric ages point to Skye, ~40 km to the North. A reconstructed eruption volume of ≥5km^3^ DRE is derived, suggesting a VEI 5 event or larger. We therefore argue, contrary to long-held perception, that large-volume silicic volcanism and its associated climatic effects were likely integral to the BPIP during the opening of the North Atlantic.

## Introduction

**Figure 1:**
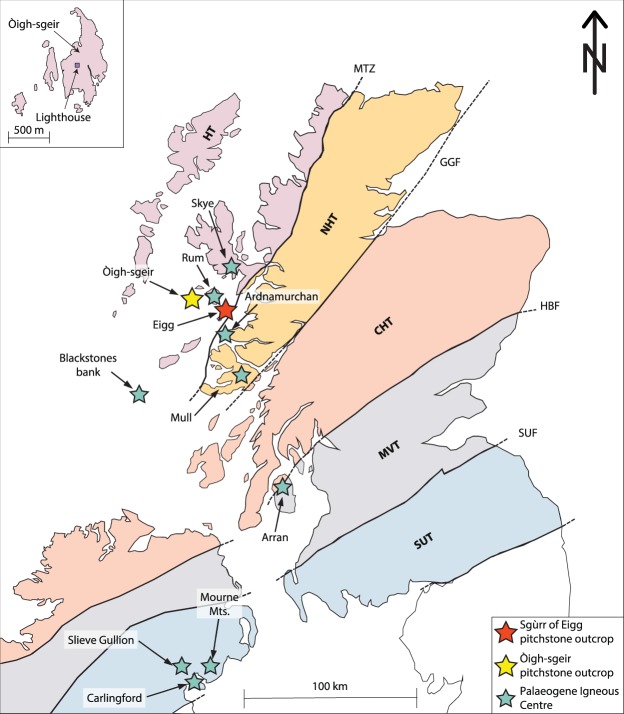
Geological overview map of Scotland with tectonic terranes and respective boundaries. Òigh-sgeir and Eigg, in the Inner Hebrides are highlighted with a yellow and a red star, respectively, while all other Palaeogene Igneous Centres are given with pale green stars. Inset top left shows the Òigh-sgeir skerries. HT = Hebridean Terrane (Lewisian gneisses, some Torridonian and Cambro-Ordovician sedimentary rocks); NHT = Northern Highlands Terrane (Moine metamorphic rocks, some Old Red Sandstone); CHT = Central Highlands (or Grampian) Terrane (Dalradian metamorphic rocks, some Moine and Old Red Sandstone); MVT = Midland Valley Terrane (Carboniferous sedimentary and volcanic rocks, some Permo-Triassic sedimentary rocks and Old Red Sandstone); SUT = Southern Uplands Terrane (Ordovician-Silurian sedimentary rocks). Faults: MTZ = Moine Thrust Zone; GGF = Great Glen Fault; HBF = Highland Boundary Fault; SUF = Southern Uplands Fault.

The basalt-dominated British Palaeogene Igneous Province (BPIP) records silicic magmatism as small plutons and intrusive sheets, as downfaulted extrusive rocks in central calderas, and as thin silicic tuffs in the associated lava fields, but large-volume pyroclastic eruptions from any of the Scottish Palaeogene centres are not known (e.g. refs^1–4^). On the one side, uplift and erosion could have obliterated the former evidence, whereas on the other side, large silicic eruptions may not have been a characteristic feature in the BPIP during the initiation of the North Atlantic (e.g. refs^5,6^). The North Atlantic Igneous Province (NAIP) records two major phases of volcanic activity, the first between 61–58 Ma, during which the bulk of the BPIP formed and a later phase between 56–54 Ma (refs^7,8^). Aerially widespread (and hence voluminous) Palaeogene silicic tuffs and ash layers from the second phase of volcanic activity are known from Greenland (e.g. ref.^9^), Denmark^10^, and the North Sea (e.g. ref.^11^). It is therefore important to establish if explosive silicic volcanism was also important during the early stages of N-Atlantic volcanism, especially in the Scottish sector (Fig. 1), or if large-explosive silicic events were not significant at this stage.

The columnar porphyritic pitchstones of the Sgùrr of Eigg [NM 460 847] and the rocky islets of Òigh-sgeir (Hyskeir) [NM 160 690] are rare examples in the BPIP of silicic Palaeogene volcanic rocks outside central complexes. The two pitchstone outcrops (>30 km apart) have previously been suggested to represent remnants of a single large silicic eruption (e.g. refs^12–15^), but the most recent compositional data for the Sgùrr of Eigg, do not match those of the Òigh-sgeir pitchstone (cf. refs^15,16^; Supplementary Information S1, Fig. S1). Therefore, there is now a pressing need to systematically test if these two outcrops represent remnants of a single large eruption or not.

## Geological Background And Earlier Investigations

**Figure 2:**
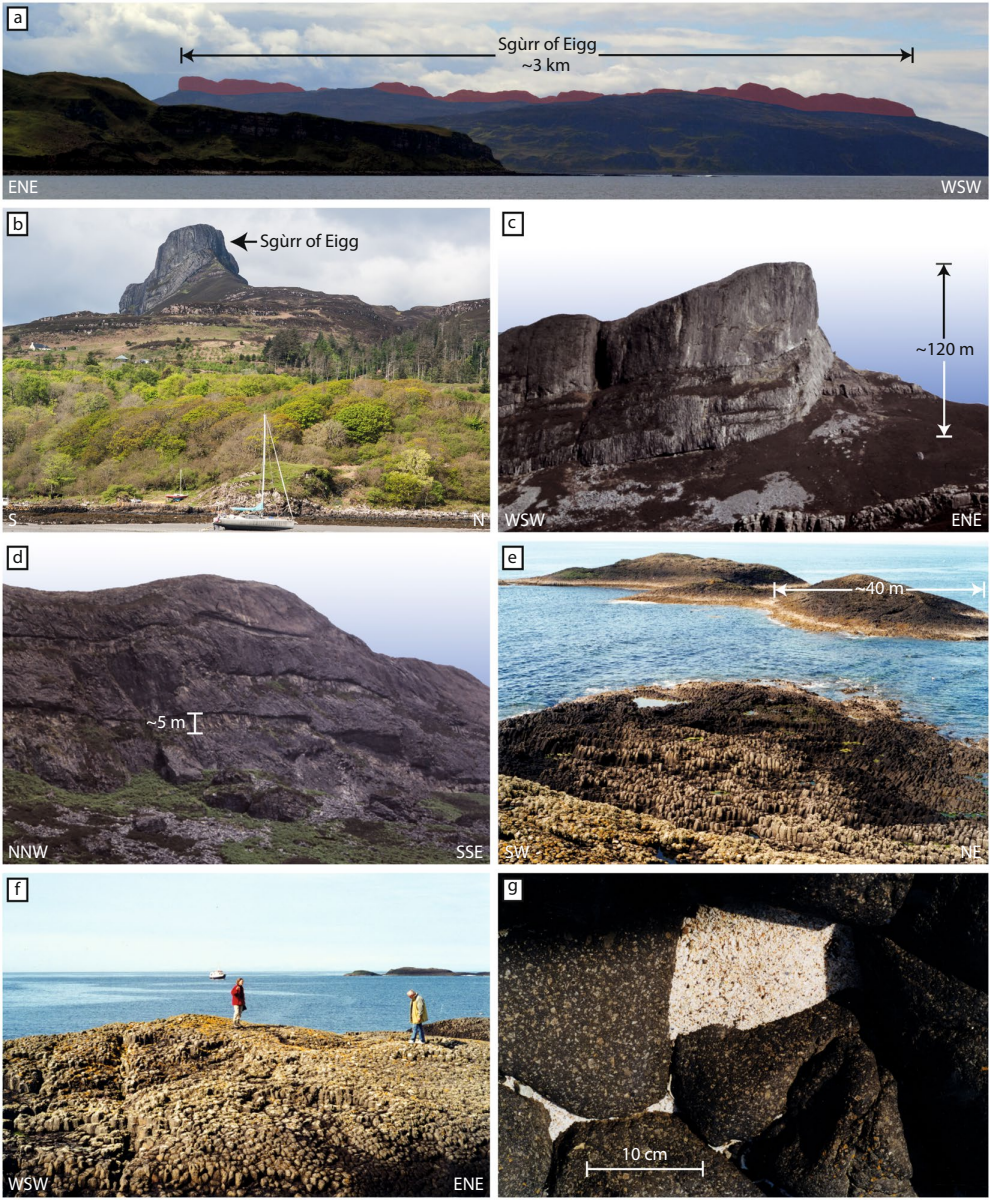
Field pictures of the Òigh-sgeir and Sgùrr of Eigg pitchstones. (**a**) Sgùrr of Eigg Pitchstone ridge viewed from the north (highlighted in red). The northern part of the Isle of Eigg is seen in the foreground. (**b**) An Sgùrr, the easternmost point of the Sgúrr of Eigg pitchstone viewed from the SE. The Sgùrr of Eigg ridge consists of columnar jointed pitchstone and stretches over the southern part of the Isle of Eigg. (**c**) An Sgùrr, viewed from the south. (**d**) Close up of the southern face of the Sgùrr of Eigg ridge. Note the pale sheets in the otherwise dark grey pitchstone. (**e**,**f**) Columnar jointed pitchstone at the Òigh-sgeir skerries. Furthermore, a cave along the trace of the post-emplacement fault offsets the skerries and exposes submarine extensions of the columns. (**g**) Close-up of the Òigh-sgeir pitchstone, showing plan view onto polygonal cooling columns.

The Sgùrr of Eigg dominates the south of the Isle of Eigg^17–19^ (Fig. 2). The Sgùrr of Eigg was initially interpreted as a lava flow that occupies a steep-sided valley floored by a fluvial conglomerate^17^. In contrast, Harker^13,20^ considered the pitchstone as intrusive, but Bailey^21^ supported Geikie’s lava flow interpretation. Subsequently, Allwright^14^ and Emeleus^15^ showed that the basal metre of pitchstone (e.g. at Bidean Boideach [NM 4412 8667]) is a welded vitric tuff, implying a pyroclastic origin, whereas the pale-coloured felsite sheets on the southern face of the Sgùrr (Fig. 2) were regarded as intrusive^14,15^. However, the most recent account of the Sgùrr of Eigg and its underlying conglomerate^16^, interprets the columnar jointing, weathering patterns and sharp undulating boundaries within the pitchstone to represent several rapidly emplaced ignimbrites from a low but sustained pyroclastic column. The pervasive, base-parallel flow-banding and associated recumbent isoclinal folds are held to indicate post-depositional ‘lava-like’ rheomorphism. The authors also argue, on the basis of similar mineralogy and chemical compositions between the dark pitchstone and the pale sheets on the southern face, that the latter represent devitrified zones at the tops of successive flow pulses, rather than separate intrusions. Further, Brown and Bell^16^ tentatively proposed that the Sgùrr of Eigg pitchstone was sourced from Skye and envisage, a formerly extensive ignimbrite sheet. This is, however, uncommon and no such enormous outflow sheet has hitherto been recognized in the BPIP. A possible extension to the Sgùrr of Eigg pitchstone was recently suggested by Smith^22^, who identified a 5 km long, sinuous submarine ridge south of the Isle of Muck, but to date no material is available from this locality. An early age of 52.1 ± 1.0 Ma (Rb-Sr) for the pitchstone^23^, was subsequently revised to 58.72 ± 0.07 Ma (Ar-Ar)^24^.

Òigh-sgeir is a group of rocky islets located ~8 km SW of Canna and >30 km WNW of Eigg and it is exclusively made of columnar porphyritic trachyte pitchstone (e.g. refs^12,13^) (Figs 1 and 2). The petrography of the Òigh-sgeir pitchstone was investigated by Judd^18^, Geikie^17^, Harker^13^, Allwright^14^, and Emeleus^15^ who all agreed that there were strong structural, petrographic and geochemical similarities between the Òigh-sgeir and the Sgùrr of Eigg. Brown and Bell^16^, however, recently provided data that show the Sgùrr of Eigg major-element composition to differ from the available Òigh-sgeir pitchstone data (Fig. S1). Notwithstanding these differences, Brown and Bell^16^ support the Sgùrr of Eigg and Òigh-sgeir pitchstone to be part of a single eruption (see ref.^15^) and on the basis of available isotope data from the Sgùrr of Eigg, they propose a source within the Hebridean Terrane. Specifically, they argue for the 40 km-distant Skye Central Complex, invoking a very large silicic eruption. Given the possible importance of large volcanic eruptions for preparing the conditions that ultimately led to the Palaeocene-Eocene Thermal Maximum (PETM) at ~56 Ma (refs^8,25,26^), it is important to establish the magnitude of silicic eruptions in this part of the North Atlantic Igneous Province in the run up to the PETM.

To ultimately test if the Sgúrr of Eigg and Òigh-sgeir pitchstones represent the remnants of a single large silicic pyroclastic eruption, we compare the Òigh-sgeir and Sgùrr of Eigg pitchstones by employing newly acquired crystal, whole rock, and radiogenic and stable isotope data. In addition to testing for a common ancestry of the Sgùrr of Eigg and Òigh-sgeir pitchstones, we also use the new data to evaluate if a specific source within the Western Red Hills of Skye is plausible. If the pitchstones of Eigg and Òigh-sgeir can be shown to represent remnants of a single large eruption, it would be one of the largest as yet recorded from the North Atlantic Igneous Province (NAIP) and certainly the largest known in the British Palaeogene Igneous Province.

## Assessing a Common Ancestry

While using mineralogy and major and trace elements to assess consanguinity of different bodies of rock may potentially be compromised in porphyritic rocks due to modal mineral variations or variable degrees of fractionation, isotopic tracers may be more reliable. This is because radiogenic isotopes are insensitive to physical changes such as temperature, pressure or crystallisation conditions, but reflect the various source compositions involved in petrogenesis (e.g. ref.^27^). Stable isotopes, in contrast, do change with physical conditions, but fractionation factors are not very large at magmatic temperatures^28^. To establish a common ancestry between the Eigg and Òigh-sgeir pitchstones a central theme of our approach is that a positive match for radiogenic and stable isotopes is ideally coupled with a positive match for mineral types, -textures, and -composition, and also with a match for whole rock and groundmass major element trends.

As regards radiogenic isotopes, four tectono-stratigraphic terranes are traversed through the British Palaeogene Igneous Province, from Skye and Rum in the North, to Carlingford and the Mourne Mountains in the South^3^ (Fig. 1; Supplementary Information S2). These terranes are isotopically so diverse, that ascending mantle-derived magmas have been variably affected by the specific terrane through which they have erupted (cf. refs^23,27,29–31^). The Sgùrr of Eigg and Òigh-sgeir pitchstones lie within the Hebridean Terrane which is characterised by Archaean Lewisian basement (e.g. refs^23,27^), and is separated by the Moine thrust from the Northern Highlands Terrane where additional isotopically-distinct Moine-type metasedimentary rocks occur (e.g. refs^29,30^). This led Dickin and Jones^23^ to postulate a source within the Hebridean Terrane for Sgùrr of Eigg. Isotope data from Òigh-sgeir have so far not been available.

### Field descriptions

**Figure 3:**
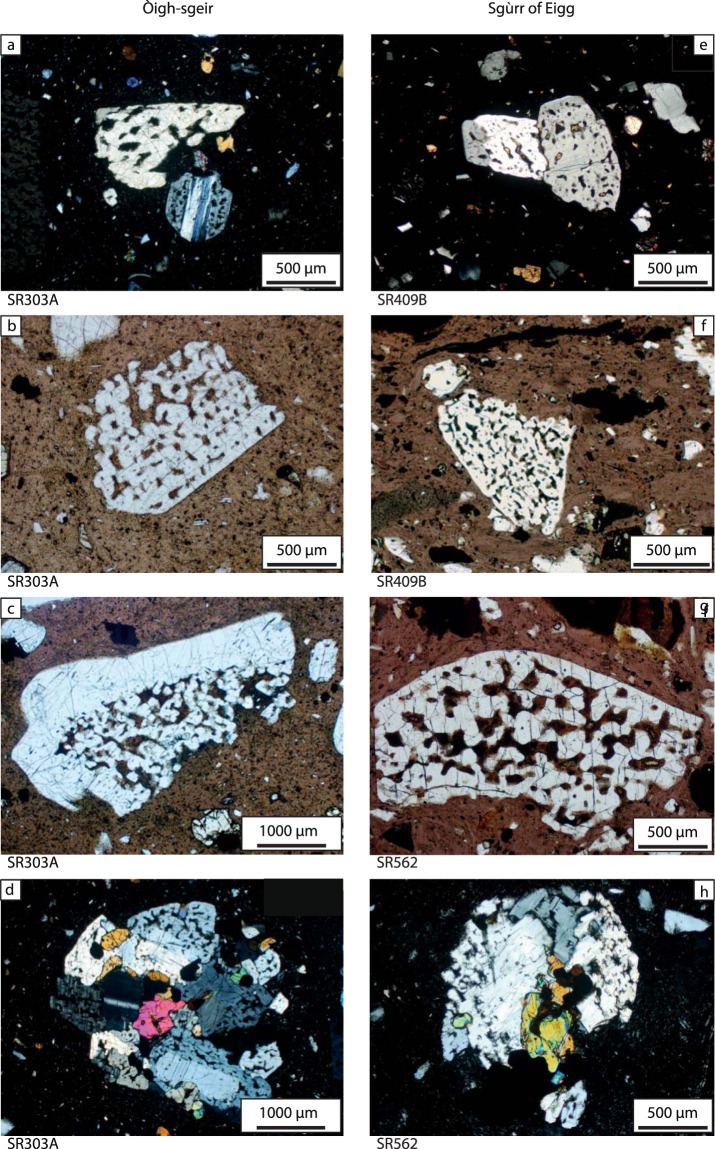
Comparison of textures in single crystal and crystal clots in the Òigh-sgeir and Sgùrr of Eigg pitchstones. (**a**) Microphotograph of Òigh-sgeir pitchstone with resorbed feldspar and small pyroxene grains in glassy matrix (CPL). (**b**) Òigh-sgeir pitchstone with large resorbed feldspar crystal (PPL). (**c**) Large (sieve-textured) and resorbed feldspar crystals in glassy Òigh-sgeir pitchstone. Note the outer edge (top side of the crystal) is intact, while resorption appears to have attacked the feldspar “internally” (PPL). This ‘internal’ resorption features in the K-feldspar and Na-feldspars suggest that heating was the main agent for resorption^75^. (**d**) Representative plutonic inclusion (plagioclase, pyroxene, opaques) in Òigh-sgeir pitchstone (CPL). Note the resorbed feldspar in the inclusions. (**e**) Small plagioclase cluster in Sgùrr of Eigg pitchstone (CPL). Note the resorption of the feldspars. (**f**) Base of Sgùrr of Eigg pitchstone. Note the mafic schlieren (‘fiamme’) in top part of image (above resorbed feldspar), indicating mafic magma was present during the eruption (PPL). (**g**) Photomicrograph of sieve-textured feldspar fragment in Sgùrr of Eigg pitchstone with intact outer edge (upper side), set in a glassy groundmass (PPL). (**h**) Photomicrograph of upper Sgùrr of Eigg deposit with a plutonic inclusion that comprises resorbed feldspar, pyroxene and opaques. Most of the feldspars show evidence of initial to advanced resorption. Importantly, the Sgùrr of Eigg and Òigh-sgeir pitchstones are strikingly similar, displaying the same mineral-types, mineral assemblage and identical mineral textures.

**Figure 4:**
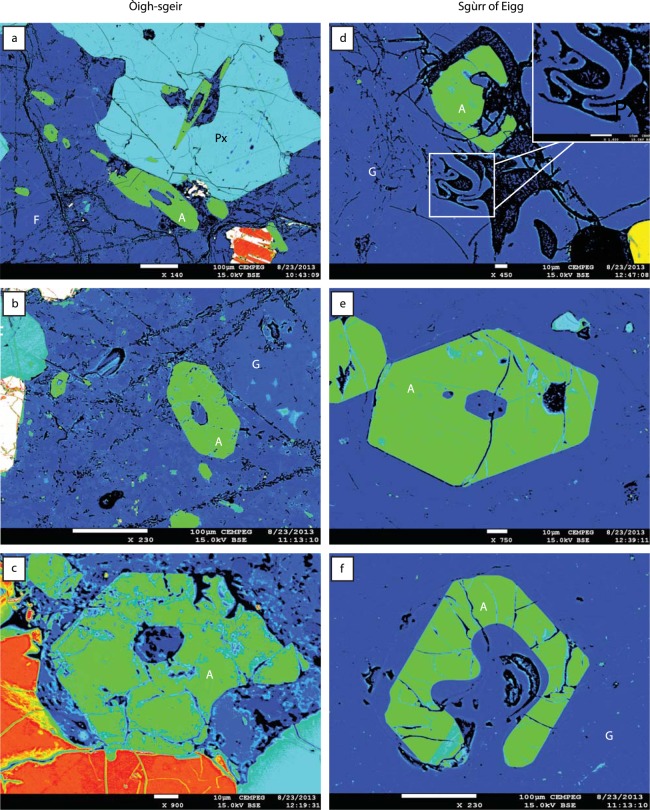
False colour BSE images of apatite crystals in Òigh-sgeir and Sgùrr of Eigg pitchstone deposits. (**a**) A plutonic inclusion in Òigh-sgeir pitchstone with feldspar (F), pyroxene (Px), apatite (green) and oxide (red-white). Note the various cuts of apatite crystals frequently show hollow interiors (skeletal growth). (**b**) Apatite in matrix glass (G), Òigh-sgeir pitchstone. Note the smaller euhedral apatites also. (**c**) Margin of a mineral clot in Òigh-sgeir pitchstone where an apatite crystal is becoming detached from an oxide (red) and is already largely surrounded by matrix glass. Note the rounded inner channel and the lobate embayment on the crystals lower right. (**d**) Dendritic skeletal apatite crystal (green) in glassy matrix in the Sgùrr of Eigg pitchstone. Also note the welded glass streaks below the ‘A’ in this image (also enlarged in inset), implying re-agglutinated fiamme as a result of rheomorphic flow in a hot and plastic condition (cf. ref.^16^). (**e**) Euhedral free-floating apatite in Sgùrr of Eigg pitchstone. (**f**) Free-floating apatite in pitchstone glass; Sgùrr of Eigg. The apatite shows euhedral outer crystals faces, but is breached and resorbed in its interior, indicating late-stage dissolution of the mineral.

The Òigh-sgeir skerries are made exclusively of pitchstone^13^ and are situated on the basaltic Canna Ridge, a south-westwards submarine extension of the Skye Lava Group^15^. Columnar jointing can be seen in tidally exposed cliffs (Figs 1 and 2) and a post-emplacement fault offsets the skerries. The Eigg pitchstone, in turn, is very well exposed along a 3 km long, steep-sided ridge extending north-westwards from the Sgùrr of Eigg to Bidean Boideach (Fig. 2). There, a west-facing cliff exposes an underlying conglomerate that was filling a former steep-sided valley in the Eigg Lava Formation^15^. At the Recess [NM 4606 8462], an alcove formed by erosion below the massive pitchstone in the southeastern part of the Sgùrr of Eigg, a steep face exposes spectacularly-jointed pitchstone overlying the conglomerate with large boulders of red Torridonian sandstone and plant remains, including the ‘Eigg pine’^13^. This conglomerate was widely viewed as fluvial^12,14,15,21^ and an entire system of fluvial palaeo-valleys connected to the former Sgùrr of Eigg valley has been postulated^14^. The basal pitchstone is strongly brecciated and thoroughly mixed with the underlying sedimentary material. While Emeleus^15^ suggested the occurrence of phreatic explosions, Brown and Bell^16^ consider it a peperite. In addition, the lowest ~10 cm of the Sgùrr of Eigg pitchstone lack the otherwise abundant large feldspar crystals, but contain numerous small crystal fragments and millimetre-sized wispy basaltic fiamme in a vitreous, closely-packed matrix (Figs 3 and S2). Most recently, the Sgùrr of Eigg pitchstone was interpreted as a welded ignimbrite deposit that formed from a pyroclastic density current, and the underlying conglomerate as a debris flow^16^. The presence of mafic fiamme and re-agglutinated former glass shards documents a previous fragmented stage for these rocks, and thus corroborates an overall pyroclastic mode of formation (Figs 3 and 4).

### Petrography of the pitchstones

Here we provide a summary of previous petrological descriptions^14–16^ and our own observations on the investigated rock samples.

The Sgùrr of Eigg pitchstone has a black, vitreous to dark matt-grey appearance on fresh surfaces and contains between 20 and 35% of ‘free-floating’ crystals of plagioclase (≤15%), anorthoclase (~10%), Fe-rich clinopyroxene (~2%), orthopyroxene (≤3%), Fe-Ti oxides, accessory apatite (~2%), and traces of sulphides. Notably, the rock is devoid of quartz, but fine-scale flow banding is common (Fig. 3). The Òigh-sgeir pitchstone is also black to dark brown and semi-vitreous with abundant mm-sized feldspar crystals (plagioclase and anorthoclase), plus minor augite and orthopyroxene set in pale-brown glass (no quartz present). The matrix contains microcrystals of plagioclase, oxides, sulphides, and accessory apatite (Figs 3 and 4). ‘Flow banding’ has locally been observed. The larger ‘free-floating’ feldspars are characterised by pronounced resorption textures in both the Òigh-sgeir and Sgùrr of Eigg pitchstone samples (Fig. 3). These are pronounced in the interiors of crystals, but notably not on outer crystal surfaces. The crystal assemblage in the Òigh-sgeir pitchstone is therefore extremely similar to that in the Sgùrr of Eigg and, moreover, the approximate proportions of minerals appear very similar too (Figs 3 and 4). In the basal, fragmental portion of the Sgùrr of Eigg, the occurrence of basaltic fiamme is noted (Figs 3f and S2), implying hot, mafic magma was involved in the eruption^32,33^.

### Petrography of plutonic inclusions

Both pitchstone groups contain abundant plutonic, broadly monzonitic, inclusions that host an almost identical mineral assemblage to that seen ‘free-floating’ in the glassy groundmass (Fig. 3). The plutonic inclusions are characterised by holocrystalline mineral arrangements of feldspar, pyroxene, oxide, apatite and sulphide crystals. Resorption features are frequent, and include distinctly resorbed feldspar and apatite in both pitchstone groups and in their respective plutonic inclusions (Figs 3 and 4). Apatite is particularly noteworthy as it shows late resorption textures superimposed on original skeletal growth textures in all pitchstone and plutonic inclusion samples investigated (Figs 4, S3 and S4, Supplementary Information S3). The identical mineralogical and textural crystal assemblage implies that the plutonic inclusions are the source for the crystals in the Òigh-sgeir and the Sgùrr of Eigg pitchstones and, moreover, the abundance of plutonic inclusions with disintegration textures suggests a remobilised monzonitic pluton or crystal mush as the main source of the crystals and inclusions (cf. ref.^34^).

### Major elements

**Figure 5:**
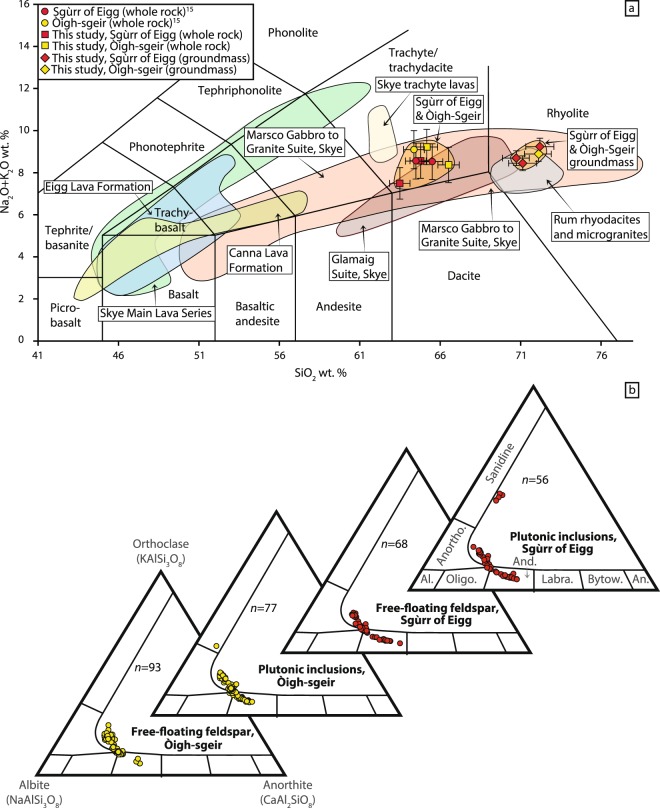
Geochemical data for the Òigh-sgeir and Sgùrr of Eigg pitchstones and minerals. (**a**) Total alkalis vs. silica diagram of the ‘OSSEP’ whole rock, glass and groundmass compositions. The Òigh-sgeir and Sgùrr of Eigg pitchstones show a narrow compositional cluster for whole rocks and another narrow compositional group for groundmass (glass) compositions. The Canna Lava Formation on Rum and Canna belongs to the Skye central complex, while the Eigg lavas pre-date Rum and Skye (see Supplementary Information S1 and Supplementary Table S6 for data sources and selection). Notably the combined OSSEP data overlap with the mixed-magma Marsco gabbro-granite suite of Skye and plot close to known trachyte lavas from the Skye Lava Group, while differing markedly from the rhyodacites and microgranites exposed on Rum. (**b**) Feldspar compositional triangles (An-Ab-Or) for Òigh-sgeir “free-floating” feldspar in the pitchstone (bottom left), feldspar in Òigh-sgeir plutonic inclusions (second from left), free-floating feldspar in Sgùrr of Eigg pitchstone (third from left), and from plutonic inclusions in the Sgùrr of Eigg pitchstone (top right). Note the compositional ranges are virtually identical. The plutonic inclusions in both pitchstone groups show some sparse K-rich compositions that are not present in the free-floating feldspar populations. This indicates that K-rich feldspar that was liberated from the plutonic inclusions was dissolved in the pitchstone melt (cf. ref.^37^).

The Òigh-sgeir and Sgùrr of Eigg samples reported by Emeleus^15^ and those used in this study form tight clusters for the whole rock and for the groundmass data on a total-alkali vs. silica diagram (Fig. 5a; Supplementary Table S1), classifying the whole rocks as trachyte due to the absence of quartz (see also Supplementary Information S1; Supplementary Table S2). The overlap is striking, and a common origin is possible. The combined Òigh-sgeir and the Sgùrr of Eigg whole rock suite (which we term ‘OSSEP’) does not overlap with known felsic rocks from Rum, the closest major volcanic centre to the two outcrops (Fig. 1) and only the OSSEP groundmass data show a composition that is overlapping the whole rock data of the Rum felsic suite. The OSSEP samples are therefore not matching with the Rum felsic suite. The OSSEP whole rock and groundmass data do, however, overlap with the broad compositional range of the Marscoite Hybrids and the Glamaig granites in the Western Red hills on Skye and, moreover, the OSSEP whole rock suite plots close to the trachytic lava flows of the Skye Lava Group^35,36^ (Fig. 5a).

#### Feldspar compositions

Oligoclase to anorthoclase feldspar is the dominant mineral in the Òigh-sgeir and Sgùrr of Eigg pitchstones. The Òigh-sgeir feldspar crystals show a compositional range from An_16_ to An_38_ (*n* = 93) for larger isolated (i.e. free-floating) feldspar crystals and An_17_ to An_37_ for plutonic feldspar (*n* = 77). The Sgùrr of Eigg feldspar crystals show An_18_ to An_45_ for free-floating feldspar (*n* = 68) and An_13_ to An_41_ for plutonic feldspar (*n* = 56) (Fig. 5b; Supplementary Table S3). The compositional spectrum of all four groups thus overlaps.

Notably, anorthoclase is present in both plutonic feldspar groups, but is sparse in the free-floating feldspar populations of both pitchstones. In the plutonic inclusions, euhedral plagioclase cores are frequently mantled by highly resorbed anorthoclase. This implies that preferential dissolution of low Ca-anorthoclase occurred, indicating re-heating of the pitchstone magma, because low Ca-feldspar has a lower melting temperature than Ca-rich plagioclase (cf. refs^34,37^).

### Oxygen isotopes

**Figure 6:**
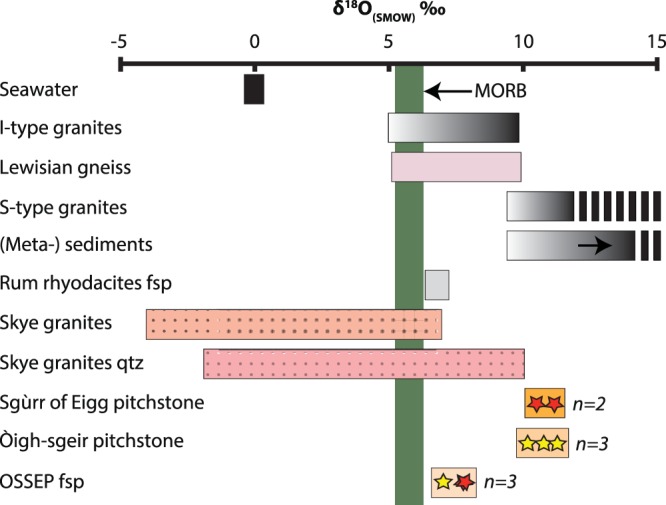
Oxygen isotope whole rock and feldspar crystal data of Sgùrr of Eigg and Òigh-sgeir pitchstones in comparison to known reference compositions. Fields for seawater, I- and S-type granites, Lewisian gneiss, Rum rhyodacite feldspar, Skye granites, Skye granite quartz (data from crystal interior) and global metasediments are given for reference. The Òigh-sgeir and Sgùrr of Eigg results overlap within the analytical uncertainty of the method. The data are, moreover, elevated relative to mantle values^28^, implying crustal input to the OSSEP magmas. See text for details and Supplementary Table S6 for data sources.

Five whole rock samples and three larger feldspar crystals, from the Òigh-sgeir and Sgùrr of Eigg pitchstones were analysed for oxygen isotopes (Fig. 6; Supplementary Table S4). The Sgùrr of Eigg whole rocks show δ^18^O values from +10.5 to +11.2‰ (±0.2) (*n* = 2), whereas the Òigh-sgeir whole rock samples yield δ^18^O values from +10.2 to +11.3‰ (±0.2) (*n* = 3). The two pitchstone groups’ whole rock values thus overlap within the uncertainties of the method. The Sgùrr of Eigg feldspar crystals (*n* = 2) show δ^18^O values from +7.75 to +7.8‰ (±0.3), and the Òigh-sgeir crystal (*n* = 1) shows a δ^18^O value of +7.04 (±0.3). To put these results into context, mantle values are around +5.7 (±0.3) and the global sedimentary field ranges from 9 to ≥30‰ (refs^28,38^). Because closed system fractional crystallisation can raise mantle values by ~1‰ (ref.^28^), the OSSEP δ^18^O feldspar values imply an open magmatic system, likely involving contamination by local Lewisian basement (which is up to +10‰, refs^39,40^). The OSSEP whole rocks record very high values and they straddle the boundary between S- and I-type granites at ~+10‰ (e.g. ref.^41^). However, although late contamination of the melt that now constitutes the glassy groundmass is possible, post-eruptive alteration of the glassy groundmass is quite likely^42,43^. Irrespective of the exact cause of the high δ^18^O whole rock data, the oxygen isotopes record an identical magmatic (crystal separates) and post-eruptive (whole rock) history of the Sgùrr of Eigg and the Òigh-sgeir pitchstones.

### Radiogenic isotopes

**Figure 7:**
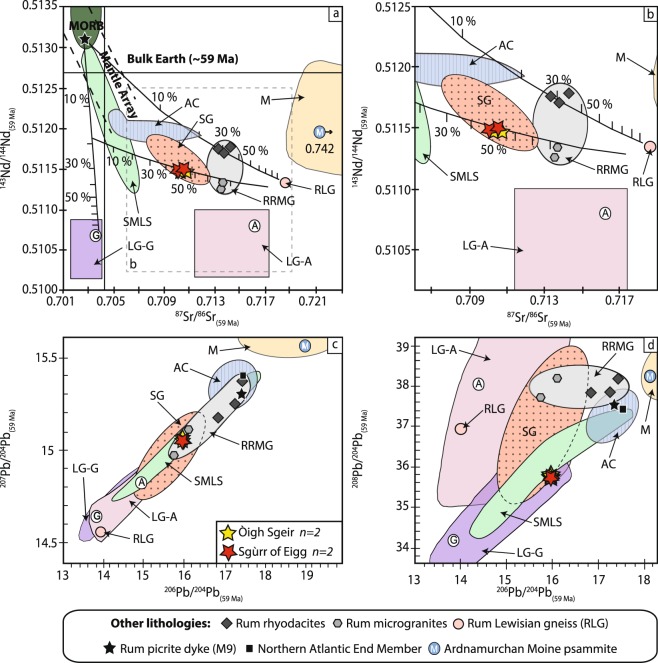
Sr, Nd and Pb isotope ratios for the Sgùrr of Eigg and Òigh-sgeir pitchstones. (**a**,**b**) ^143^Nd/^144^Nd vs. ^87^Sr/^86^Sr, (**c**) ^207^Pb/^204^Pb vs. ^206^Pb/^204^Pb, and (**d**) ^208^Pb/^204^Pb vs. ^206^Pb/^204^Pb (at 59 Ma). The average crustal terrane compositions of Lewisian gneiss (granulite- and amphibolite-facies, LG-G and -A) and Moine meta-sedimentary rocks (M), as well as upper mantle proxies are given for reference. Fields for the major igneous centres in the region are shown for comparison: Skye granites (SG), Skye Main Lava Series (basalts-hawaiites-benmoreites; SMLS), Rum rhyodacites and microgranites (RRMG), and Ardnamurchan cone sheets (basalts-andesites-rhyolites, AC). An initial trend towards Lewisian granulite-facies material is implied by the Sr-Nd data and confirmed by the Pb isotopes. The OSSEP samples plot where the granulite-facies and amphibolite-facies Lewisian gneiss fields overlap, which is remote from the field for the relatively radiogenic Moine rocks and the Palaeogene mantle. To investigate the specific contamination history of the OSSEP samples, we can use the primitive picritic dyke from the Isle of Rum (‘sample M9’) or the average North Atlantic mantle End Member, which are both widely viewed as approximations of the isotope compositions of Palaeocene mantle-derived magmas. The OSSEP Sr and Nd data are consistent with a mixing model whereby the Sgùrr of Eigg and Òigh-sgeir magmas are derived from upper mantle melt that underwent 10–20% contamination by lower crustal granulite-facies-type Lewisian gneiss. This first contamination event was followed by up to 50% mixing/contamination with upper crustal amphibolite-facies type Lewisian Gneiss^23,27,31,44^. The felsic rocks of the Sgùrr of Eigg and Òigh-sgeir pitchstones thus record identical cumulative effects of contamination events in the deep and in the shallow crust. See Supplementary Table S6 for data sources.

Sr, Nd and Pb isotope data for the Sgùrr of Eigg and the Òigh-sgeir pitchstones (corrected to 59 Ma, ref.^24^) as well as for representative Rum microgranites are presented in Fig. 7a–c and Supplementary Table S4. This, to the best of our knowledge, includes the first radiogenic isotope data for the Òigh-sgeir pitchstone. Figure 7a–d shows the new data together with data for relevant igneous and crustal lithologies from the Hebridean and Northern Highlands terranes. The age-corrected isotopic ratios of the Sgùrr of Eigg and the Òigh-sgeir pitchstones show a very narrow range for ^87^Sr/^86^Sr (0.710047 to 0.710732), ^143^Nd/^144^Nd (0.511483 to 0.511522), and for ^206^Pb/^204^Pb, ^207^Pb/^204^Pb, and ^208^Pb/^204^Pb (15.947 to 15.973, 15.057 to 15.072 and 35.785 to 35.831, respectively), and the radiogenic isotope data thus overlap within the uncertainties of the analyses. Given that the Palaeogene Igneous Centres are known to show changing degrees of assimilation with time (e.g. refs^29,31,37,39^), a large time gap between Sgùrr of Eigg and Òigh-sgeir pitchstones is not supported by the radiogenic isotope data. Instead, a single and common source for the Òigh-sgeir and Sgùrr of Eigg pitchstones is implied.

## Establishing the Eruption Source

The petrographic, chemical, and isotopic characteristics of the Sgùrr of Eigg and Òigh-sgeir pitchstones broadly overlap in all lines of investigation and we conclude that the two outcrops, although >30 km apart, derive from the same source. They represent the remnants of a regionally extensive large-magnitude explosive silicic eruption. Our results now fully vindicate previous suggestions for a large, regionally extensive silicic eruption in the BPIP^12,14–16,21^.

To establish the exact eruption source of the OSSEP tephra, a more detailed discussion is required. The prerequisites for the OSSEP source include (i) evidence of silicic magmatism together with a major heat source capable of generating the silicic magmas through fractionation and crustal assimilation, (ii) chemical and isotopic compatibility between the OSSEP and the source, (iii) evidence of silicic-mafic magma mixing, and (iv) comparable ages between the OSSEP pitchstones and the potential source.

The crustal units of the Hebridean and the NW-Highland terranes of NW-Scotland have isotopic signatures that are distinct from each other and from Palaeogene mantle compositions (Figs 1 and 7) and isotopic characteristics have successfully been used to fingerprint contamination and volcanic sources within the BPIP mafic and felsic units (e.g. refs^30,33,44–46^). In the Hebridean Terrane, ascending Palaeogene magmas encountered granulite- and amphibolite-facies rocks of the Lewisian complex (2.5 Ga) exclusively, whereas in the Northern Highland Terrane, in addition to Archaean Lewisian rocks, Proterozoic metasediments of the Moine series (1.0 Ga) are present^3^. The rhyodacites from the Isle of Rum^31,33^, and the microgranites of the Western Red Hills of Skye^27,35^, are characterised by the typical Hebridean Terrane isotopic signature^23,27,47^. In contrast, the rhyolites in the composite cone sheets of nearby Ardnamurchan show Lewisian contamination closely followed by uptake of Moine lithologies^23,30^. The Òigh-sgeir and the Sgùrr of Eigg pitchstones indicate a common and solely Lewisian (Hebridean) contamination history and the OSSEP rocks are isotopically distinct from the felsic Palaeogene rocks of the NW-Highland Terrane (as e.g. recorded in the magmatic trends from Ardnamurchan and Mull; cf. refs^23,29,30^) (Fig. 7). However, the silicic rocks from Rum and Skye are similar to the OSSEP suite in that they are characterised by solely Lewisian contamination histories^23,27,30,31,33^.

The Pb isotope data also exclude Moine contamination, because the Sgùrr of Eigg and Òigh-sgeir pitchstones are much less radiogenic than the Moine meta-sedimentary rocks or the assumed mantle in the region (e.g. ref.^48^). The radiogenic isotope data presented thus support the initial observation of Dickin and Jones^23^ that the Sgùrr of Eigg pitchstone magma experienced Lewisian crustal input only and hence must be sourced from the Hebridean Terrane, a conclusion that now applies to the Òigh-sgeir pitchstone also. However, although Dickin and Jones^23^ and Emeleus^15^ favoured an eruption centre for the Sgùrr of Eigg magma on, or very close to the island of Eigg, Brown and Bell^16^ advocated a single regionally extensive pyroclastic deposit and suggested Skye to the North as the source area. Voluminous silicic magma that formed dominantly from crustal anatexis is most effectively produced near a large heat source^31^, yet there is no large gravity anomaly below Eigg^49,50^. Assuming one of the nearby large igneous centres with (i) a strong positive gravity anomaly, and (ii) a characteristic ‘Hebridean Terrane’ isotope signal to be the source for the OSSEP eruption, then indeed Rum and Skye are the only two realistic options (cf. refs^23,33^).

Both the Rum and, especially, the Skye central complexes have major silicic components and both have strong positive Bouguer (gravity) anomalies indicative of underlying mafic intrusions^49,51^. Notably, however, the Rum rhyodacite and microgranite samples do not match with the OSSEP data for major elements or Sr, Nd and Pb-isotope compositions and different degrees of fractionation and crustal assimilation are recorded for the Rum rhyodacites and microgranites compared to the OSSEP suite (Figs 5, 6, 7; Supplementary Tables S1 and S4). For a specific contamination history of the OSSEP suite, see Supplementary Information S4. Rum therefore does not appear to provide a fit. On the other hand, we note that the OSSEP radiogenic isotope data consistently overlap with the isotope composition of the Red Hills granitoids on Skye^23,47^. This is in accord with major element similarities, including overlap with the mixed-magma Marsco granite suite, the erupted Skye trachyte lavas, and the mixed magma Glamaig Granite on Skye^35,36^ (Figs 5 and 7).

Regarding oxygen isotopes, feldspar crystals from the Rum rhyodacites have δ^18^O values of ~+6.9‰ (ref.^52^) and the granites on Skye have whole rock δ^18^O values of −4 to +7‰ (refs^53,54^). In both, the Òigh-sgeir and the Sgùrr of Eigg pitchstones we find disequilibrium between phenocrysts and whole rock and we attribute the disequilibrium to post-eruptive exchange of the glassy groundmass with external fluids, which can shift δ^18^O values by as much as 10‰ (ref.^42^). The feldspar crystals are, in turn, more likely to reflect pristine magmatic values (e.g. ref.^43^). This aspect strengthens the proposed relationship between the Òigh-sgeir and Sgùrr of Eigg pitchstones, implying an identical δ^18^O history for the two pitchstones. Lastly, the OSSEP feldspar δ^18^O values overlap with δ^18^O values of (alteration-resistant) quartz grains from the Western Red hills granites and the least hydrothermally altered Skye granitoids^54,55^, while they differ from δ^18^O in the feldspars from the Rum rhyodacites^52^ (Fig. 6).

A final aspect is the geochronology of the region. The Rum igneous complex and its rhyodacites and microgranites are older than 60 Ma (between 60.3 and 60.5 Ma, refs^52,56^) and can therefore not be the source of the OSSEP suite. The radiometric ages from Skye, however, compare favourably with the available radiometric age of the Sgùrr of Eigg pitchstone. The Loch Ainort Granite on Skye, for instance, has an Ar-Ar age of 58.58 ± 0.13 Ma (ref.^57^), while the Sgùrr of Eigg pitchstone has an overlapping Ar-Ar age of 58.72 ± 0.07 (ref.^24^). In addition, there is petrographic evidence of silicic-basic magma-mixing in the Skye granites of Glamaig and of Marsco in the Western Red Hills (e.g. refs^35,36,47^). The available age constraints, combined with petrography, major elements and isotope data, thus argue strongly for Skye and especially the Western Red Hills as the most probable source for the OSSEP suite.

## Assessing the Magnitude of the OSSEP Event

**Figure 8:**
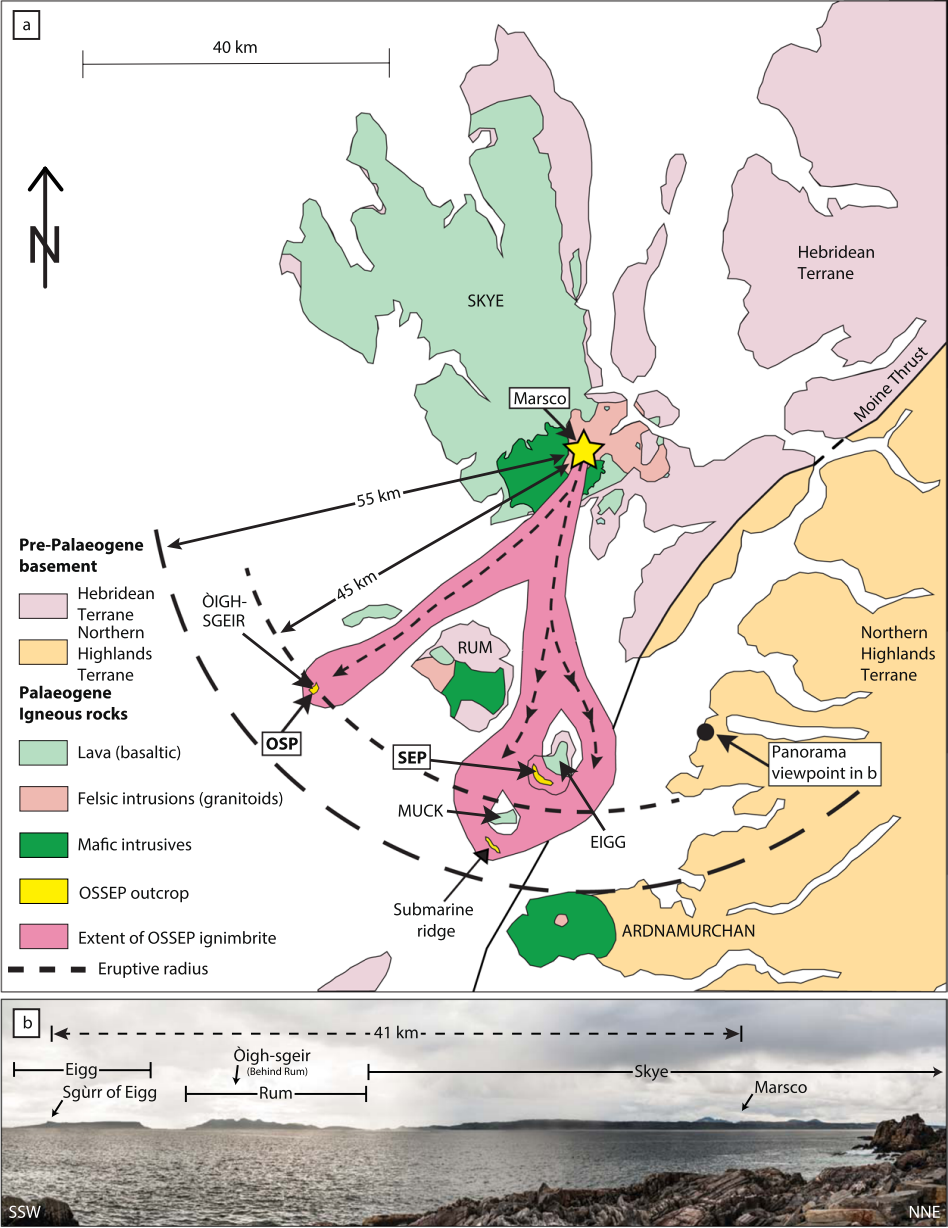
Source and run-out distances of the OSSEP event. (**a**) OSSEP outcrops (in yellow), including the recently detected submarine ridge south of Muck, define the potential area affected and allow an estimate the minimum runout distance of the ‘OSSEP pyroclastic event’. The vent area on Skye is defined on isotopic, geochronological, and geological grounds (see text for details). (**b**) Panorama photograph of Eigg, Rum and southern Skye. The Sgùrr of Eigg and the inferred source of the OSSEP eruptives (Marsco) are marked on the photo. Viewpoint (Portnaluchaig) is marked with filled black circle in (**a**).

The ground between Skye and Òigh-sgeir was traversed by rivers in the Palaeogene that were in part filled with debris flows from Rum, as indicated by erosional clasts from Rum that are found in conglomerates within the lava flows of southern Skye and Canna^15,58^. Coupled with Moine-type quartzite pebbles detected in the SW-Skye conglomerates, a topographically low-lying palaeo-landscape between Skye and Rum is indicated (Fig. 8). At the time of the OSSEP eruption this river-dissected low-lying landscape (e.g. refs^14,15^) contrasted with the older Rum edifice that was a likely significant topographic high. Accepting Skye as the vent location and Rum as an older and upstanding edifice, the exposures on Eigg and Òigh-sgeir ought to be viewed as two separate but co-eruptive flow lobes (Fig. 8). Linear run-out distances for eruptions from Skye (e.g. from the Marsco ring-dyke or the Glamaig granite) to Òigh-sgeir and Eigg would be ~45 and 41 km, respectively, which are not unreasonable for high-temperature pyroclastic density currents (e.g. ref.^59^). Since pyroclastic flows are usually “ground-hugging”, we would expect that the OSSEP deposit preferentially filled valleys and depressions. Because the OSSEP deposit is currently only preserved as former valley-fills distal to its assumed source, the original deposit must have been filling low ground elsewhere and was thus likely of considerable volume with presumably major effects on the landscape and biota of the region. The maximum established eruptive radius of 50 km (distance from Marsco to the marine ridge south of Muck) and the distance between Òigh-sgeir and the marine ridge (36 km, Fig. 8) allow calculation of an area of up to 1010 km^2^ that was directly affected by the OSSEP event. Although, the OSSEP rock volume removed by erosion was likely significant (e.g. refs^15,24^) we have no information on ignimbrite-filled river lengths, over-bank deposits or distal ashes (cf. ref.^59^) (Fig. 8). Estimation of the magnitude of this ancient eruption is thus difficult. Attempting a first-order estimate for the OSSEP eruptive volume, we can use the distances from Skye to Eigg (~41 km) and to Òigh-sgeir (~45 km), together with the pitchstone cross-section on Eigg on the face exposed at Bidean Boidheach (~150 m in width, ~120 m in thickness). Underlying the Sgùrr of Eigg are conglomerates, giving the valley a U-shape^15,16^ and U-shaped cross-section area, with total former valley width of 300 m was therefore chosen (cf. ref.^15^), but we have also considered possible wider ones of 500 m and 750 m (Fig. S5). The minimum volumes using 300 m valley widths is 1.4 km^3^ for a lobe from Marsco to Eigg and 2.5 km^3^ for Marsco to Òigh-sgeir (550 m wide valley). These two lobes combine to 3.9 km^3^ (dense-rock equivalent; DRE) and translates to a magnitude 5 on the Volcanic Explosivity Index (VEI). When an overbank facies is considered and a probably more realistic meandering river system, with for example ~80 or 100 km valley length (as indicated by field geology), the projected volume of the combined OSSEP event exceeds 10–15 km^3^ of erupted material (Supplementary Table S5) and may reflect a VEI magnitude 6 eruption or higher.

Although, these projected volumes are a first-order approximation, the derived magnitude of the OSSEP eruptions compares with historical examples such as the prolific 1991 Pinatubo eruption (~5 km^3^ DRE) or the infamous 1883 Krakatau eruption (11 to 15 km^3^ DRE)^60,61^, two of the largest eruptions in historical times. In the absence of data for the CO_2_ and sulfur contents of the pitchstone magma, the climatic effects of the OSSEP event are difficult to estimate. To constrain the maximum CO_2_ released from the OSSEP eruption we used comparable data from flood basalt eruptions of similar size as the OSSEP event (5 to 10 km^3^ eruptive volume), which range between 0.7 and 0.14 Gt CO_2_ (e.g. ref.^62^). These values are small compared to annual anthropogenic CO_2_ emissions of ~6.3 Gt/year in the late 20^th^ century^63^ and since the mass fraction of CO_2_ in the melt is likely less in a felsic magma due to solubility relations^64^, the effects from magmatic CO_2_ released by the OSSEP eruption were likely limited.

Assuming that the sulfur content in the OSSEP magmas was similar to those of silicic eruptives elsewhere (cf. ref.^65^), the OSSEP eruption could have ejected some 1*10^14^ g of sulfur into the atmosphere (see Supplementary Information S6). This amount of sulfur is about an order of magnitude higher than recorded in historical VEI 5 and 6 events. Due to the short degassing time-scales and large amounts of sulphur released into the atmosphere during an explosive felsic eruption (cf. ref.^66^), the OSSEP eruption could have caused direct climate and environmental effects for several years after the eruption in form of acid rains and by atmospheric cooling as a result of aerosol formation (cf. refs^25,66^).

Coupled with the occurrence of widespread ash beds in E-Greenland, Denmark and the North Sea as well as with eroded ignimbrite vents elsewhere in the BPIP^4,9–11,33,67,68^, our results imply that large-scale explosive silicic eruptions have likely been common during both phases of volcanic activity during the opening of the North Atlantic, including the British Palaeogene Igneous Province. This realisation paints a more violent picture of the rift to drift transition of the North-Atlantic region between 61 and 56 Ma than previously assumed. Moreover, the identification of the OSSEP event implies that ash-beds of this event, and likely similar events of the first phase of NAIP, may be present elsewhere, but poor resistance to weathering and erosion resulted in relatively few examples of this silicic volcanism being preserved outside the central complexes. Although individual silicic eruptions may have had only temporary climate effects, latest research indicates that several events and sources of volatiles are required to explain the build-up of climate active volatiles towards the Palaeocene-Eocene Thermal Maximum at ~56 Ma^8,26,69,70^. We therefore argue that the collective effects of repeated silicic volcanism from Palaeogene magmatic centres exposed in Scotland, Ireland and Greenland and those nowadays submerged in the N-Atlantic (e.g. the Blackstone bank) may have provided a so far underappreciated contribution towards the severe climate effects around the Palaeocene-Eocene boundary.

## Methods

### Samples

Samples SR303A and B (Òigh-sgeir pitchstone) and SR490, SR562 (Sgùrr of Eigg pitchstone) were selected from the sample catalogue in the BGS Memoir on Rum and the Small Isles^15^. The Sgùrr of Eigg pitchstone samples were collected by C.H. Emeleus and both outcrops are described in detail in Emeleus^15^. Reference specimens of these samples can be inspected with the British Geological Survey (www.bgs.ac.uk).

### Major and trace elements

Major and trace element analyses were undertaken on the Sgùrr of Eigg pitchstone samples SR490 and SR562 and on Òigh-sgeir pitchstone samples SR303A and SR303B. Samples were analysed on a Spectro X-Lab EDP XRF at the University of St. Andrews. Analyses were carried out on fused beads and all analyses were performed with a Rh tube. Calibration was performed using international geological reference standards. The data are compared to the range of available compositions from these and related localities (e.g. refs^14–16,35,36^).

### BSE imaging, Element maps and EMPA analysis

BSE images, Element maps and quantitative mineral data were acquired using the field emission source JEOL JXA-8530F Hyperprobe at the Department of Earth Sciences, Uppsala University, Sweden. The run conditions for wavelength-dispersive quantitative analysis were 15 kV accelerating voltage and 10 nA probe current with 10 s on peak and 5 s on lower and upper background. Groundmass and glass analyses in the OSSEP samples were carried out in either 4 × 4, 5 × 5, 6 × 6 and 7 × 7 grids using a 10 or 15 µm diameter defocused electron beam. This resulted in 16 to 49 analyses per individual grid. The step size between every single analysis in the grid was 20 or 30 µm and the analysed area in a single grid was thus between 100 × 100 µm to 230 × 230 µm. The analyses were subsequently averaged for the respective sample and normalized. Spectrometers 1 and 2 used TAP crystals to analyse Na, Al and Si, Mg respectively. Spectrometer 3 collected Ca and Mn with PETJ crystal. Spectrometer 4 analysed K and Ti with PETJ crystal. Iron and Cr were measured by LIFH on spectrometer 5. The PRZ correction is routinely used. The following standards were used for calibration; wollastonite for Ca and Si, pyrophanite (MnTiO_3_) for Mn and Ti, magnesium oxide for Mg, orthoclase for K, albite for Na and aluminium oxide for Al, Fayalite for Fe and chromium oxide for Cr. Detection limits are ≤150 ppm for all major elements. Analytical precision was measured on Smithsonian Institute mineral standards. Augite (USNM122142, n = 220) showed uncertainties of ≤2.1% s.d. for SiO_2_, MgO, CaO, FeO and Al_2_O_3_. Anorthite (USNM 137041, n = 430) has reproducibility of ≤1.1% s.d. for SiO_2_, CaO and Al_2_O_3_ and Ca-plagioclase (USNM 115900, n = 240) showed uncertainities of ≤1.4% s.d. for SiO_2_, CaO and Al_2_O_3_.

### Radiogenic isotope analysis

Sr, Nd, and Pb isotope analyses were conducted on two whole-rock samples from the Sgùrr of Eigg pitchstone (SR490, SR562) and two from the Òigh-sgeir pitchstone (SR303A and SR303B) (Supplementary Table S3). The analyses were performed at the Scottish Universities Environmental Research Centre (SUERC), East Kilbride, UK, following procedures as outlined in Meyer *et al*.^31^. Sr and Nd samples were analysed on a VG Sector 54–30 thermal ionization mass spectrometer. ^87^Sr/^86^Sr was corrected for mass fractionation using ^86^Sr/^88^Sr = 0.1194. Repeat analysis of the NIST SRM-987 Sr standard gave 0.710257 ± 18 (2 s.d., *n = *14) for the duration of this study. ^143^Nd/^144^Nd was corrected for mass fractionation using ^146^Nd/^144^Nd = 0.7219. During the course of this study the SUERC internal JM Nd laboratory standard gave ^143^Nd/^144^Nd = 0.511501 ± 12 (2 s.d.). Pb was separated using standard HBr-HCl anion exchange techniques, and measured on a Micromass IsoProbe MC-ICP-MS (e.g. ref.^71^). External reproducibility of the ^206^Pb/^204^Pb, ^207^Pb/^204^Pb and ^208^Pb/^204^Pb isotopic ratios is 0.2% (2 s.d.), and analytical blanks were <1 ng. All isotope ratios have been age-corrected to 59 Ma (ref.^24^).

### Stable isotope analysis

Oxygen isotope ratios in whole rock and mineral samples were acquired using a conventional silicate extraction line (for whole rocks) and a laser fluorination line (for crystals), combined with a Thermo DeltaXP mass spectrometer at the University of Cape Town, South Africa. Approximately 10 mg of powdered sample was dried in an oven at 50 °C and degassed under vacuum on a conventional silicate extraction line attached to externally heated Ni vessels at 200 °C (refs^41,72^). Silicates were reacted with ClF_3_ (ref.^73^), and the liberated O_2_ was converted to CO_2_ using a hot platinised carbon rod. For analytical details of the laser line see Vennemann and Smith^74^. The results are reported in standard δ-notation, where δ = (R_sample_/R_standard_ − 1) × 1000 and R = ^18^O/^16^O. Samples were run in tandem with duplicate samples of the internal quartz standards (MQ) which calibrates the raw data to SMOW (Standard Mean Ocean Water; e.g. ref.^72^), and is equivalent to V-SMOW using a δ^18^O value of 10.1 for MQ (calibrated against NBS-28). During the course of this study, the analytical error for δ^18^O is estimated to be ± 0.2‰ (2σ) for whole rocks and ± 0.3‰ (2σ) for mineral analysis, based on long-term duplication of MQ.

## Electronic supplementary material

Supplementary Information

Supplementary Information 2

## Data Availability

All data generated or analysed during this study are included in this published article (and its Supplementary Information files).
