# Supplementary material for: A large explosive silicic eruption in the British Palaeogene Igneous Province
Source: Sci Rep. 2019 Jan 24;9:494. doi: 10.1038/s41598-018-35855-w (PMC6345756; doi:10.1038/s41598-018-35855-w)
Supplement: Supplementary file 1 — Supplementary Information [file 41598_2018_35855_MOESM1_ESM.pdf]

A large explosive silicic eruption in the British Palaeogene Igneous Province

Valentin R. Troll, C. Henry Emeleus†, Graeme R. Nicoll, Tobias Mattsson, Robert M. Ellam, Colin H. Donaldson, Chris Harris

Supplementary Information

Contents

Supplementary Tables
Table S1. Major and trace element composition of Òigh-sgeir and Sgùrr of Eigg Pitchstone samples.
Table S2. Major and trace element groundmass composition of Òigh-sgeir and Sgùrr of Eigg Pitchstone samples.
Table S3. Plagioclase compositions of the Òigh-sgeir and Sgùrr of Eigg Pitchstone samples.
Table S4. Sr, Nd, Pb, O isotopic ratios for Òigh-sgeir and Sgùrr of Eigg Pitchstone samples.
Table S5. Volume estimates of the OSSEP eruptive event
Table S6. Data sources for figures 5a, 6, 7

Supplementary Text
S1. Major element composition
S2. Regional geology
S3. Detailed petrographic comparison of the Òigh-sgeir and Sgùrr of Eigg pitchstones
S4. Detailed contamination history
S5. Volume estimation of the OSSEP eruptive event
S6. Possible climate effects of the OSSEP eruption

Supplementary Figures
Figure S1. Total alkalis-silica diagram of the OSSEP rocks.
Figure S2. BSE images of free-floating apatite Sgùrr of Eigg pitchstone and Òigh-sgeir pitchstone samples.
Figure S3. BSE images of plutonic inclusions Sgùrr of Eigg pitchstone and Òigh-sgeir pitchstone samples.
Figure S4. BSE image and Element maps of mafic schlieren ‘fiamme’ in the base of Sgùrr of Eigg pitchstone.
Figure S5. Possible valley shapes and dimensions filled by OSSEP eruptives based on Sgùrr of Eigg field observations.

Sample	*SR 303a*	*SR 303b*	*SR 303**	*SR 490*	*SR 562*	*HE 7406**	*EA1**	*SR333**	*SR486**
Location	**Òigh-sgeir**	**Òigh-sgeir**	**Òigh-sgeir**	**Sgùrr of Eigg**	**Sgùrr of Eigg**	**Sgùrr of Eigg**	**Sgùrr of Eigg**	**Rum**	**Rum**
Rock type	Porphyritic dacite pitchstone	Porphyritic dacite pitchstone	Porphyritic dacite pitchstone	Fine-grained basal ash flow	Porphyritic pitchstone	Porphyritic Pitchstone, Beinn Tighe	Porphyritic Pitchstone, east end of Cora Bheinn	Porphyritic rhyodacite East of Beinn nan Stac summit	Porphyritic rhyodacite North summit of Meall Breac
GR (all NM)	*155*	*155*	*155*	*4412*	*4512*	*448*	*4577*	*3970*	*3875*
	960	*960*	*960*	*8667*	*8531*	*869*	*8555*	*9408*	*9847*
Major elements (wt%)									
SiO_2	65.74	66.53	64.37	63.49	64.79	65.51	64.49	73.02	72.84
TiO_2	1.13	1.1	1.11	1.31	1.19	1.27	1.19	0.67	0.66
Al_2O_3	15.17	15.06	14.85	15.4	15.36	16.15	15.09	13.01	12.84
FeO	4.27	4.84	4.18	5.88	4.54	4.36	4.54	4.01	4.13
MnO	0.13	0.15	0.13	0.16	0.13	0.14	0.13	0.07	0.08
MgO	0.69	1.15	0.68	2.1	1.49	0.95	1.11	0.61	0.46
CaO	2.41	2.21	2.36	3.29	2.57	2.66	2.57	1.54	1.72
K_2O	4.75	4.25	4.45	3.29	3.71	4.05	3.71	3.45	3.18
Na_2O	4.55	4.12	4.65	4.2	4.85	4.48	4.85	4.47	4.71
P_2O_5	0.37	0.38	0.35	0.44	0.35	0.33	0.35	0.11	0.1
Total	99.21	99.79	97.13	99.56	98.98	99.9	98.03	100.96	100.72
Trace elements (ppm)									
Ba	2358	-	2665	2411	2479	2396	2841	993	1073
Co	-	-	20	-	-	-	3	2	3
Cr	<3	-	2	75	<3	23	3	3	2
Nb	20	-	31	18	20	27	31	18	15
Ni	<3	-	10	9	<3	n.d.	16	13	12
Rb	82	-	80	81	74	107	71	105	95
Sc	-	-	16	-	-	-	16	8	14
Sr	201	206	269	285	201	270	287	195	219
Y	47	-	46	44	51	46	50	34	21
Zr	555	-	586	477	589	581	593	339	308
La	71	-	76	59	72	-	77	61	30
Ce	147	-	158	128	149	-	150	118	60
Nd	79	63	78	74	80	-	73	57	28
Sm	61	11	-	60	61	-	-	-	-

Table S1: Major and trace element composition of Òigh-sgeir and Sgùrr of Eigg Pitchstone samples and for Rum rhyodacites.

Table S2: Major and trace element groundmass composition of Òigh-sgeir and for the Sgùrr of Eigg Pitchstone samples.

Sample	SR 303a	SR 488	SR 490b	SR 490c
Location	Òigh-sgeir	Sgùrr of Eigg	Sgùrr of Eigg	Sgùrr of Eigg
Groundmass	Microcrystalline	Microcrystalline	Glass	Glass
n	147	113	121	121
GR (all NM)	*155*	*4590*	*4412*	*4412*
	960	*8465*	*8667*	*8667*
(wt. %)				
SiO_2	72.17	72.27	70.85	71.21
TiO_2	0.66	0.56	0.76	0.74
Al_2O_3	14.53	14.82	14.96	14.80
FeO	2.20	1.99	2.72	2.73
MnO	0.08	0.09	0.11	0.10
MgO	0.35	0.29	0.59	0.63
CaO	1.07	0.77	1.36	1.34
K_2O	5.03	5.10	4.52	4.30
Na_2O	3.87	4.06	4.09	4.09
NiO	0.02	0.02	0.02	0.02
Cr_2O_3	0.01	0.01	0.02	0.01
V_2O_3	0.01	0.01	0.02	0.02
Total*	100	100	100	100

*All data normalised to 100.

Table S3. Representative plagioclase compositions of the Òigh-sgeir and for the Sgùrr of Eigg Pitchstone samples.

	Free-floating feldspar, Òigh-sgeir						Plutonic inclusions, Òigh-sgeir						Free-floating feldspar, Sgùrr of Eigg						Plutonic inclusions, Sgùrr of Eigg					
Sample name:	**SG 320**	**SG 232**	**SG 204**	**SG 299**	**SG 302**	**SG 235**	**SG 349**	**SG 346**	**SG 284**	**SG 263**	**SG 243**	**SG 282**	**OS 139**	**OS 171**	**OS 173**	**OS 169**	**OS 153**	**OS 149**	**OS 045**	**OS 058**	**OS 070**	**OS 007**	**OS 050**	**OS 109**
**Na_2O**	6.20	6.24	6.14	2.03	6.33	6.66	6.76	6.64	6.60	6.72	6.62	6.73	6.79	6.69	6.73	6.68	6.66	6.46	6.89	6.79	6.87	6.55	6.82	6.91
**SiO_2**	60.05	61.52	60.74	73.56	60.84	62.25	63.16	62.97	62.90	62.24	63.35	62.58	63.77	63.12	62.93	62.75	63.05	61.87	63.49	62.79	63.03	63.21	62.32	63.30
**Al_2O_3**	24.13	22.97	23.72	14.58	24.01	23.12	22.06	21.79	22.30	22.49	22.46	22.89	22.13	22.20	22.49	22.24	22.63	23.12	21.55	21.59	21.72	21.49	22.06	22.30
**MgO**	0.05	0.04	0.00	0.53	0.02	0.04	0.01	0.02	0.04	0.01	0.04	0.04	0.01	0.05	0.05	0.02	0.02	0.03	0.00	0.02	0.01	0.00	0.02	0.00
**CaO**	7.62	6.82	7.58	1.16	7.11	6.12	4.79	5.02	5.37	5.42	5.48	5.77	4.94	5.05	5.28	5.44	5.63	6.35	3.70	3.70	3.92	3.79	4.26	4.65
**MnO**	0.03	0.03	0.00	0.11	0.06	0.00	0.03	0.00	0.01	0.00	0.02	0.05	0.00	0.05	0.00	0.05	0.00	0.00	0.00	0.00	0.06	0.00	0.00	0.00
**K_2O**	0.96	1.16	1.01	3.65	1.05	1.41	2.01	1.89	1.95	1.74	1.84	1.55	2.07	1.94	1.76	1.83	1.73	1.32	3.00	2.96	3.01	2.80	2.72	2.26
**TiO_2**	0.11	0.09	0.08	0.74	0.07	0.09	0.11	0.09	0.09	0.07	0.07	0.07	0.05	0.08	0.12	0.10	0.08	0.08	0.08	0.10	0.06	0.10	0.10	0.07
**BaO**	0.19	0.23	0.23	0.22	0.18	0.34	0.58	0.57	0.42	0.53	0.53	0.43	0.63	0.47	0.55	0.51	0.49	0.31	1.00	0.98	0.85	0.94	1.04	0.68
**Cr_2O_3**	0.00	0.03	0.01	0.01	0.00	0.00	0.00	0.00	0.00	0.00	0.00	0.00	0.00	0.00	0.00	0.00	0.00	0.00	0.00	0.00	0.05	0.00	0.00	0.00
**FeO**	0.60	0.61	0.62	2.62	0.52	0.60	0.47	0.54	0.48	0.48	0.56	0.54	0.50	0.50	0.62	0.53	0.57	0.55	0.42	0.46	0.51	0.49	0.43	0.50
**Total**	99.95	99.73	100.13	99.21	100.19	100.63	99.99	99.53	100.16	99.70	100.98	100.64	100.90	100.15	100.53	100.15	100.86	100.09	100.13	99.40	100.09	99.37	99.78	100.68
**Or (mol%)**	14.7	12.4	11.2	11.1	8.9	7.6	12.3	11.7	11.8	10.5	11.1	9.3	12.5	11.9	10.7	11.1	10.4	8.0	18.1	18.1	18.0	17.6	16.3	13.6
**An (mol%)**	22.8	25.9	26.9	27.1	30.3	33.1	24.7	26.0	27.3	27.6	27.9	29.2	25.1	25.9	27.0	27.6	28.5	32.4	18.7	19.0	19.7	20.0	21.5	23.4
**Ab (mol%)**	62.6	61.7	61.9	61.7	60.8	59.3	63.0	62.3	60.8	61.9	61.0	61.5	62.4	62.2	62.3	61.3	61.0	59.6	63.2	63.0	62.4	62.5	62.2	63.0

Table S4: Isotope ratios for Òigh-sgeir, Sgùrr of Eigg and for the Rum Western Granite.

Location	Sample	$^{87}Sr/^{86}Sr$ (59Ma)	2 S.E.	Sr (ppm)	Rb (ppm)	$^{143}Nd/^{144}Nd$ (59Ma)	2 S.E.	Nd (ppm)	Sm (ppm)	$^{206}Pb/^{204}Pb$ (59Ma)	2 S.E.	$^{207}Pb/^{204}Pb$ (59Ma)	2 S.E.	$^{208}Pb/^{204}Pb$ (59Ma)	2 S.E.	U (ppm)	Th (ppm)	Pb (ppm)	$\delta^{18}O$ wr (‰)	$\delta^{18}O$ fsp (‰)
Òigh-sgeir pitchstone	Sr 303A	0.710732	0.000026	201	82	0.511483	0.000012	79	61	15.949	0.002	15.057	0.002	35.785	0.004	< 1	5	14	11.3	-
Òigh-sgeir pitchstone	Sr 303B	0.710258	0.000024	219	78	0.511491	0.000012	63	11	15.959	0.0001	15.072	0.00012	35.831	0.00013	1	4	14	10.2	7.04
Òigh-sgeir pitchstone	Sr 303C	-	-	-	-	-	-	-	-	-	-	-	-	-	-	1	4	14	10.8	-
Sgùrr of Eigg pitchstone	Sr 490	0.710047	0.000028	285	81	0.511505	0.000014	74	60	15.973	0.005	15.059	0.006	35.821	0.006	< 1	4	13	11.2	7.74
Sgùrr of Eigg pitchstone	Sr 562	0.710498	0.000036	201	74	0.511522	0.000014	80	61	15.947	0.005	15.059	0.006	35.791	0.006	< 1	5	14	10.5	7.8
Rum granite	R-WG-1	0.713734	0.000015	185	99	0.511700	0.00008	62	12	16.832	0.003	15.174	0.003	37.846	0.003	1	7	13	-	-
Rum granite	R-WG-3	0.714262	0.000010	106	133	0.511774	0.000070	31	7	17.427	0.002	15.373	0.003	38.188	0.003	1	9	13	-	-
Rum granite	R-WG-6	0.713313	0.000013	99	118	0.511750	0.00007	44	10	17.248	0.003	15.251	0.003	37.877	0.003	1	9	17	-	-

*samples were age-corrected to 59 Ma[24] and Rum samples to 60.5[52,56]. Trace element concentrations were determined by XRF and isotope dilution.

Table S5. Eruptive volume for two flow lobes.

Valley width (m)	45.5 km straight valley (Marsco - Òigh-sgeir)	41 km straight valley (Marsco - Sgùrr of Eigg)	50 km straight valley (Marsco - Muck sea ridge)	80 km long meandering valley		100 km long meandering valley	
	1 lobe (km^3)	1 lobe (km^3)	1 lobe (km^3)	1 lobe (km^3)	2 lobes (km^3)	1 lobe (km^3)	2 lobes (km^3)
300 m	-*	1.38	1.68	2.69	-*	3.37	-*
550 m	2.52	2.22	4.43	4.43	8.85	5.53	11.07
750 m	3.61	3.25	6.35	6.35	12.69	7.93	15.87

*The Òigh-sgeir pitchstone is >500 m wide in outcrop. Hence, the valley between Marsco and Òigh-sgeir was probably wider than the Sgùrr of Eigg valley.

Table S6. Data sources for figures 5a, 6 and 7.

Figure	Reference field	Reference
Figure 5a	Eigg and Canna lava formations	15
Figure 5a	Skye trachyte lavas	76
Figure 5a	Glamaig suite, Skye	35,77,78,79
Figure 5a	Marsco suite, Skye	35,36
Figure 5a	Skye Main Lava Series	80
Figure 5a	Rum rhyodacites and microgranites	15,33
Figure 6	Lewisian gneiss	39,40
Figure 6	(Meta-) sediments	28,38,41
Figure 6	S and I-type granites	41
Figure 6	Skye granites	53,54
Figure 6	Skye granites qtz	55
Figure 6	Rum rhyodacites	52
Figure 6	Seawater	81
Figure 7	Skye granites	23,47,82
Figure 7	Skye Main Lava Series	44,82
Figure 7	Rum rhyodacites	33
Figure 7	Rum micrograntes	Supplementary Table S4
Figure 7	Ardnamurchan cone sheets	30,83
Figure 7	Rum M9	31,84
Figure 7	Average North Atlantic mantle End Member	48
Figure 7	Lewisian granulite	27
Figure 7	Lewisian amphibolite	27,85
Figure 7	Moine psammite	30
Figure 7	Upper Mantle	84,86

S1. Major element rock compositions

The total alkalis-silica diagram (Fig. S1) shows the previously published data and our new data of the Òigh-sgeir and Sgùrr of Eigg pitchstone samples to overlap in the trachyte field (refs. 15; this study), while OSSEP groundmass compositions plot in the rhyolite field. Note, the term trachydacite can only be applied if quartz is conspicuous (which is not the case). The data compiled in Emeleus[15], which derive from several laboratories, overlap with our data, but the whole-rock data of Brown and Bell[16] and glass data of Carmichael[87] do not overlap with either our whole-rock data or those listed in Emeleus[15], or with the OSSEP groundmass compositions of this study (Tables S1 and S2; Fig. S1). While the data of Brown and Bell[16] appear internally consistent, they cannot be reconciled with our data or with the data of Emeleus[15] and we therefore chose not to employ the Sgùrr of Eigg analyses of Brown and Bell[16] and the glass analysis of Carmichael[87].

Moreover, the combined Òigh-sgeir and the Sgùrr of Eigg sample suite does not overlap with known felsic rocks from Rum, the closest major volcanic centre to the two outcrops. In turn, the compositional overlap with the Marscoite Hybrids in the Western Red hills on Skye and the close compositions to the known trachytic lava flows of the Skye Lava Group suggest Skye as the likely source of the OSSEP eruption, rather than Rum, although, Skye is only the next closest centre to Rum[35,36,76,77,78].

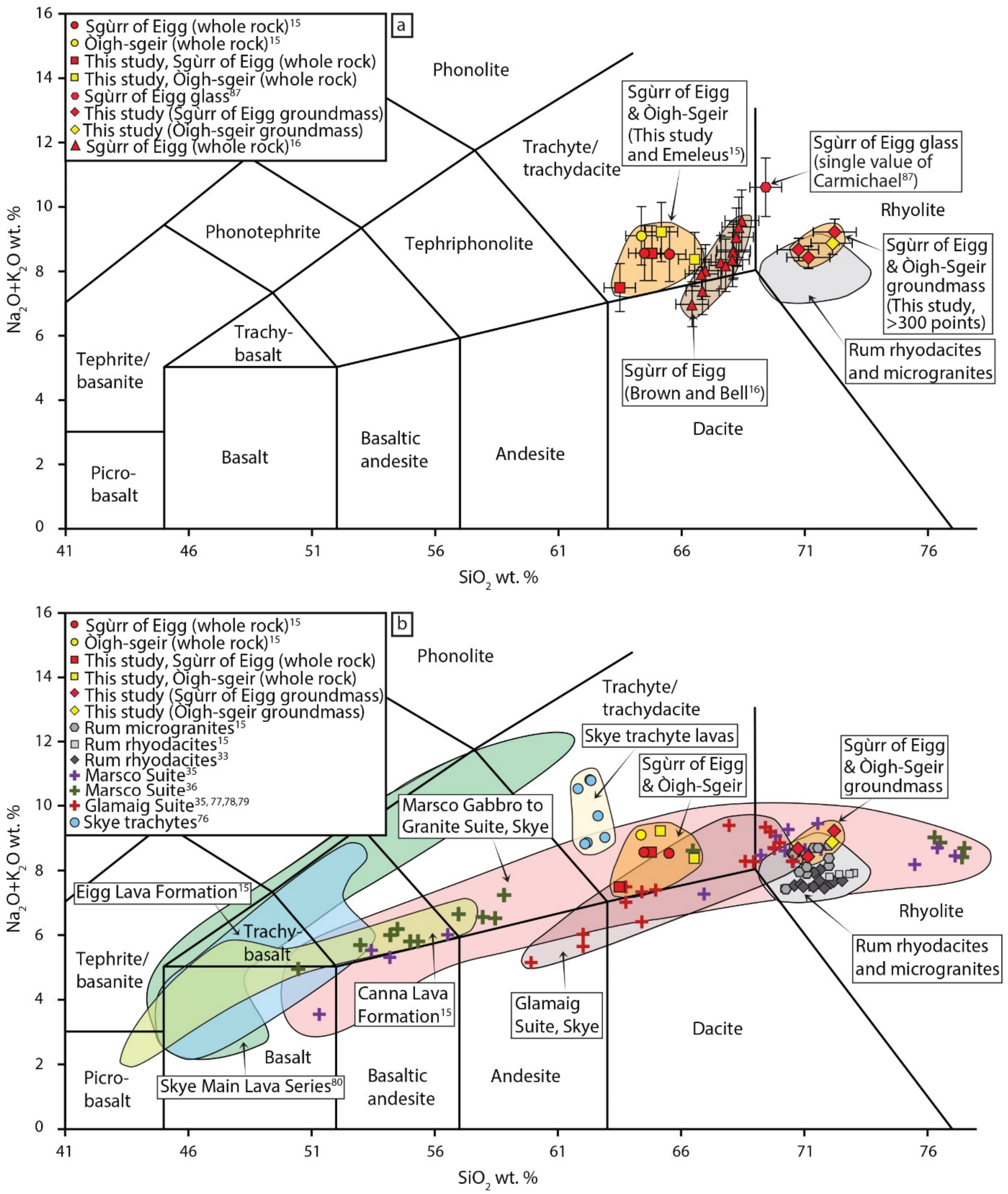

Figure S1: Total alkalis-silica diagram of the OSSEP rocks. a) the OSSEP whole rock, glass and groundmass compositions (data from this study and refs. 15, 16, 87), Rum rhyodacites and microgranites[15,33](Table S1). The data of Brown and Bell[16] and Carmichael[87] cannot be reconciled with ours or with that of Emeleus[15]. While they appear internally consistent, these data are not considered reliable for correlation purposes. b) Full comparison with regionally established compositions, e.g. Glamaig suite on Skye[35,77,78,79], Marsco suite on Skye[35,36], Skye trachyte lavas[76], Eigg and Canna lava formation[15], and the Skye Main Lava Series[80].

S2. Regional geology

Four major tectono-stratigraphic terranes are traversed on a transect through the British Palaeogene Igneous Province, from Skye and Rum in the North, to Carlingford and the Mourne Mountains in the south[3]. These crustal terranes comprise Archaean Lewisian basement in the Hebridean terrane (e.g. refs. 23, 27, 30) separated by the Moine thrust from the Northern Highlands Terrane (Fig. 1), which contains additional Moine-type upper crustal rocks (e.g. refs. 29, 30, 83). To the south, the Northern Highlands are bounded by the Great Glen Fault and Proterozoic Dalradian rocks form the major upper crustal lithology in the Central Highlands Terrane (e.g. refs. 3, 24). Further south yet, Proterozoic/Phanerozoic basement hosts the igneous centres of the Mournes, Carlingford, and Slieve Gullion (e.g. refs. 32, 37, 88, 89, 90) and is limited to the south by the major crustal faults of the Iapetus Suture. These terranes are isotopically diverse, which led to the suggestion that ascending mantle-derived magmas have been variably affected by the specific terrane through which they have erupted[23,27,29,30,31,32,33,46,83,90].

S3. Detailed petrographic comparison of the Òigh-sgeir and Sgùrr of Eigg pitchstones

Optical microscopy

Optical microscopy of representative thin section views of Sgùrr of Eigg and Òigh-sgeir pitchstones are provided in Figure 3. The image 3a from the Òigh-sgeir pitchstone formation shows resorbed feldspar crystals and small pyroxene grains in a glassy matrix (CPL). Image 3b shows a sodic feldspar and a plagioclase with strong resorption features, in Sgùrr of Eigg pitchstone (CPL). Image 3c documents sieve-textured feldspar in the glassy groundmass of the Òigh-sgeir pitchstone showing an intact outer edge (on lower side) (PPL). Image 3d depicts a large resorbed plagioclase in Sgùrr of Eigg pitchstone. Note the outer edge of the crystal appears intact, while resorption indicates partial melting of the feldspars (PPL). Microphotograph 3e shows a large resorbed (sieve-textured) feldspar crystal in glassy Òigh-sgeir pitchstone. Note the outer edge (top side of the crystal) is intact, while resorption has occurred in the feldspar "internally" (PPL). Image 3f shows sieve-textured feldspar in Sgùrr of Eigg pitchstone with intact outer edge (upper side), set in a glassy groundmass (PPL). Image 3g depicts a representative plutonic inclusion with plagioclase, pyroxene and oxide minerals from Òigh-sgeir pitchstone (CPL). Note the resorbed feldspar in the inclusions. This sample is from the base of the Sgùrr of Eigg and shows mafic schlieren in the central top part of the image (above resorbed feldspar), indicating mafic magma was present in the system. Image 3h shows a remnant plutonic inclusion of feldspar intergrown with pyroxene and opaque phases from the upper Sgùrr of Eigg pitchstone (PPL). Note the plutonic fragment contains resorbed feldspar and pyroxene. The textures in the Sgùrr of Eigg pitchstone and Òigh-sgeir pitchstone are strikingly similar, with the same major mineralogical assemblage, the same mineral types and mineral forms, and with similar modal proportions.

Electron microscopy

Figure S2 shows mafic fiamme from the base of the Sgùrr of Eigg pitchstone. Note that the major element composition of the mafic fiamme is very similar to the solid basaltic (pick-up?) clast in the lower part of the elemental maps, implying that liquid basaltic magma was present and involved in the OSSEP eruption.

BSE images of "free-floating" apatite from Sgùrr of Eigg and Òigh-sgeir (cf. ref. 91) are presented in Figures 4 and S3. Image 5_SGGM shows crystals of pyroxene (Px), oxide (O), sulphide (S), and apatite (A) in glass (G). Note the skeletal apatite in the centre of the image, which is enlarged in image **24_SGGM** where yet smaller skeletal apatites are observed that frequently show internally open c-axis channels. Also note the welded glass streaks below the 'A' in this image (also enlarged in bottom left inset), implying re-agglutinated fiamme as a result of rheomorphic flow in a hot and plastic condition (cf. ref. 16). Image **12_SGGM** records a larger oxide crystal that is fractured and caught in the process of disintegrating into the surrounding melt (now glass). Note the skeletal apatite enclosed in the oxide. The apatite

shows an internal open (crystallographic) channel as well as external resorption, although, seemingly enclosed in the oxide crystal. Replacement/corrosion along the fractures in the oxide is apparent and the outer edge of the apatite crystal also appears affected locally, while it is euhedral where in contact with the glass. Heating and chemical reactions are the likely causes for the dissolution textures. Image **11.2_SGGM** shows larger pyroxene crystal with associated apatite crystals (A), set in groundmass-glass. Note the long prismatic apatite on the left of the pyroxene crystal, which shows internal resorption from the crystal tip, including a possible vapour bubble. Image **22_OSGM** is of glassy portion of the Òigh-sgeir pitchstone (OSP), and shows a number of apatite crystals in different orientation, plus a larger sulphide grain in the centre of image. The apatites exhibit their long-needle-like morphology when cut parallel to their c-axis, while a hexagonal outline is seen perpendicular to this axis. Note the frequently open interior channels of the apatite crystals. Inset shows further examples of skeletal apatites in Òigh-sgeir (glass image 4 OSGM). Image **21_OSGM** shows a single apatite grain in glassy groundmass of Òigh-sgeir pitchstone with characteristic open interior channel.

BSE images from plutonic inclusions in the Sgùrr of Eigg and in the Òigh-sgeir pitchstones are provided in Figure S4. Images **15_SGPX** and **16_SGPX** show plutonic inclusions in the Sgùrr of Eigg pitchstone. Intergrown feldspar (F), pyroxene (Px), oxides (O), and sulphides (S) are observed. Note the skeletal textures of the apatite crystals. Images **3_OSPX, 19_OSPX, 18_OSPX** and **25_OSPX** illustrate plutonic inclusions in the Òigh-sgeir pitchstone. Note the similarity in mineralogy and textures, especially the accessory phases such as apatite. Image **19_OSPX** records the process of disintegration, offering a mechanism for liberation of originally plutonic crystals into the groundmass of the OSSEP. Notably, all Òigh-sgeir plutonic inclusions contain skeletal apatite irrespective of whether infiltrated by melt (now glass; e.g. image **19_OSPX**) or massive (e.g. **25_OSPX**), while resorbed apatites appear to be present mainly among the free-floating apatite population (e.g. Figure S3), implying that the apatites were not in equilibrium with the ambient melt in which they reside.

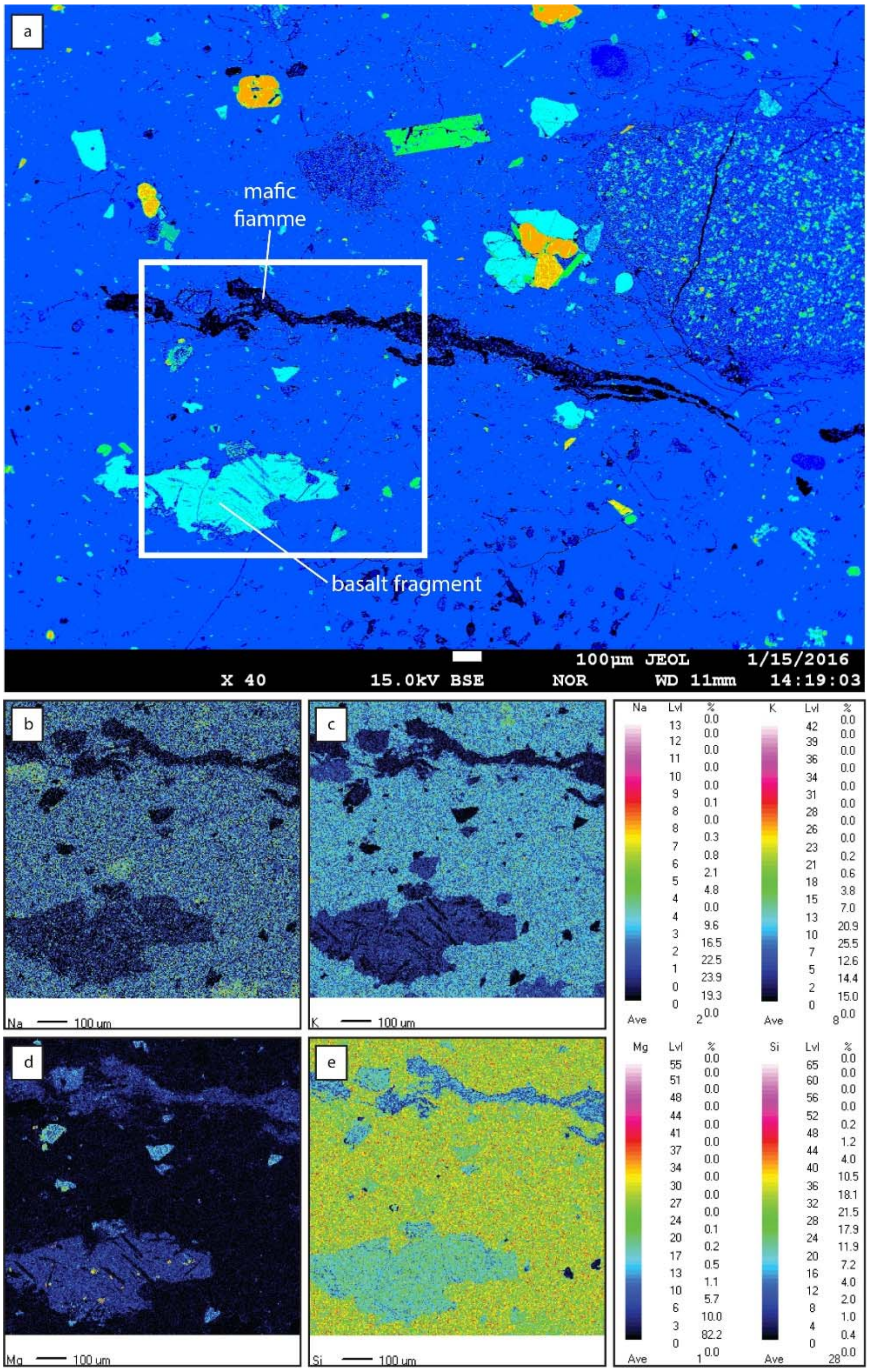

***Figure S2 a. False colour BSE image of the base of Sgùrr of Eigg pitchstone**. Mafic schlieren 'fiamme' are in central part of image (dark), while a basaltic pick up clast is highlighted in the lower left in light blue. White box in **a** indicates area of the quantitative element maps in **b-e)**. Element maps of Na, K, Mg and Si of mafic fiamme and basalt fragment in Sgùrr of Eigg pitchstone show them to be of similar composition, confirming the presence of mafic fiamme in the eruption products.*

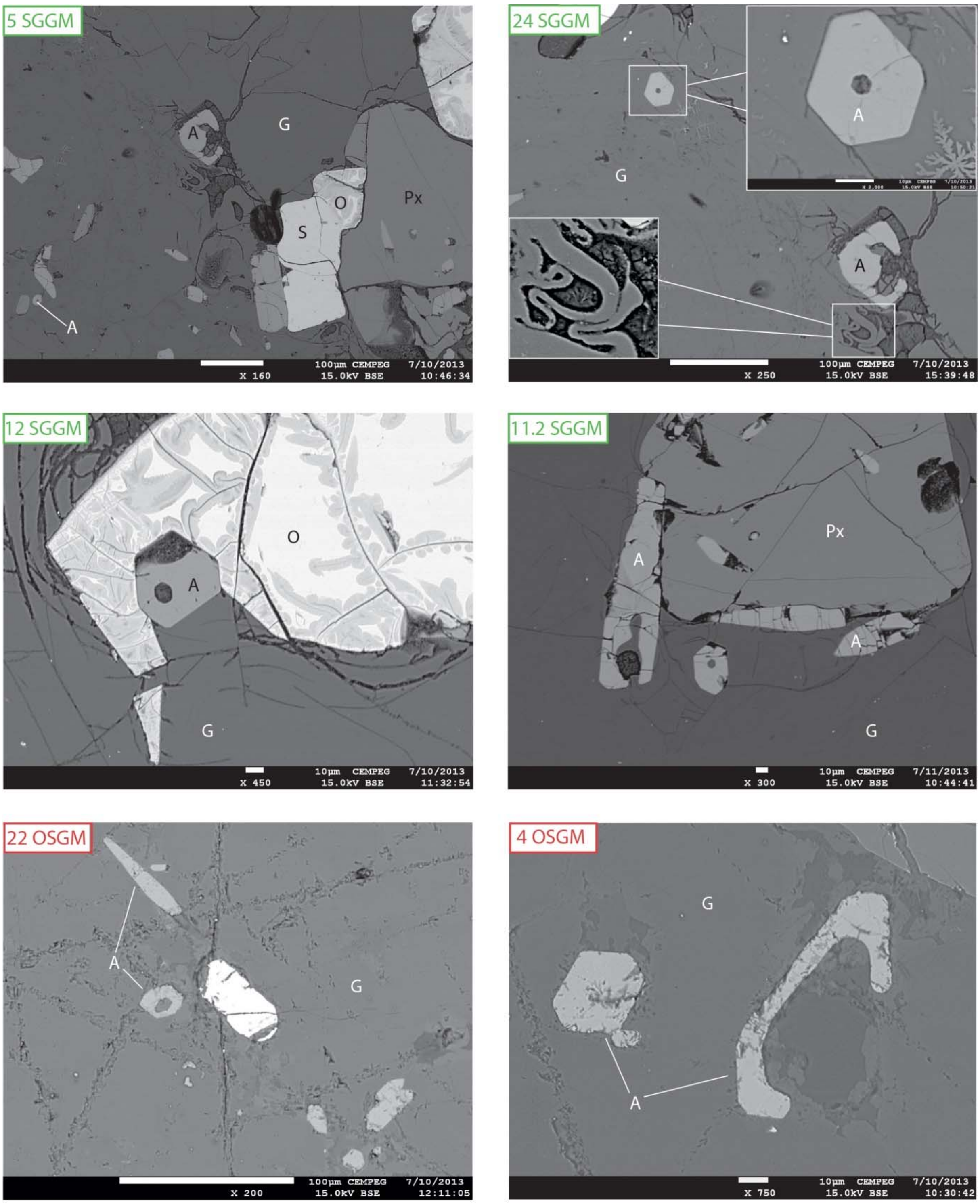

Figure S3: BSE images of free-floating apatite Sgùrr of Eigg pitchstone and Òigh-sgeir pitchstone samples. *See supplementary text for details.*

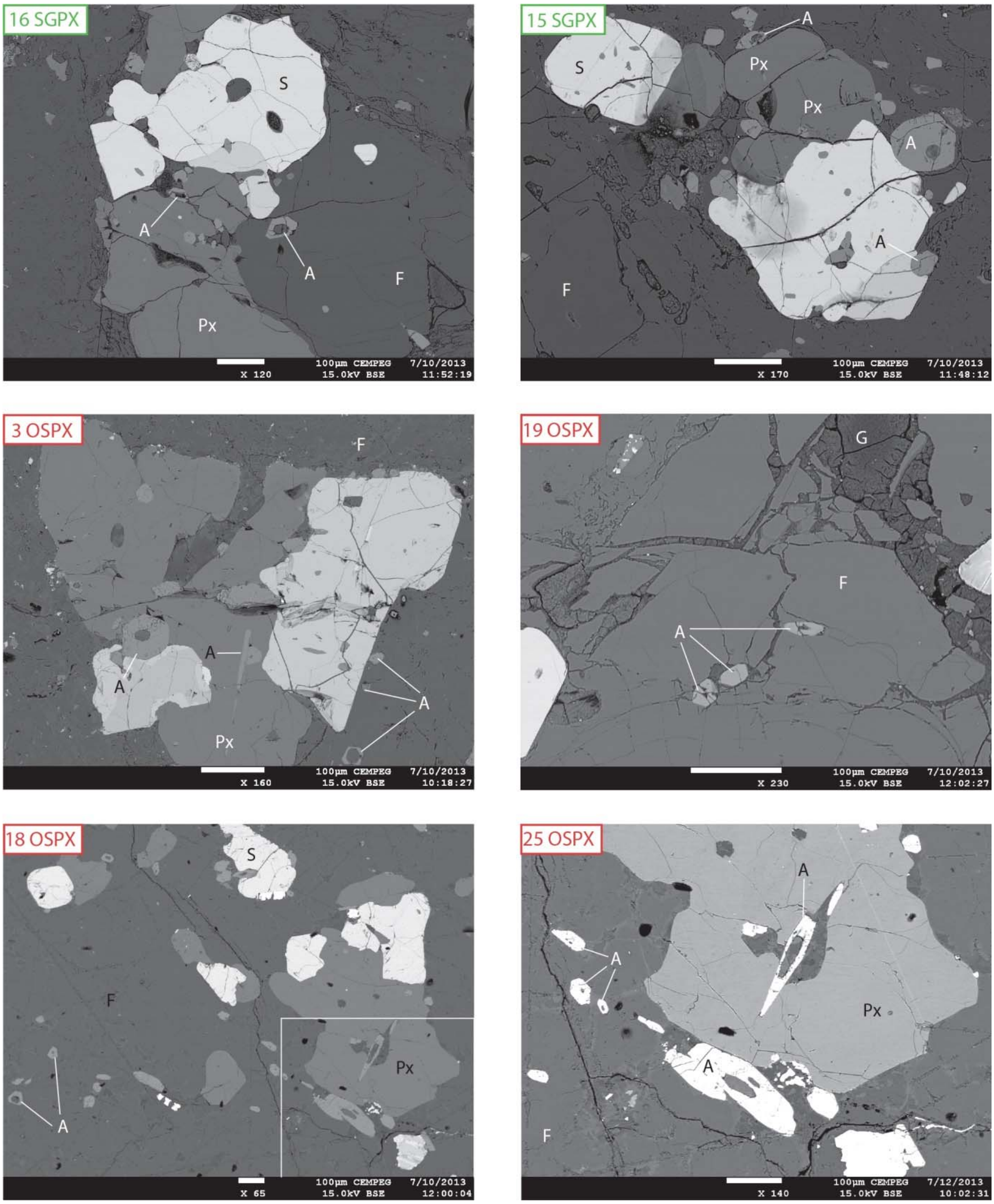

***Figure S4: BSE images of plutonic inclusions Sgùrr of Eigg pitchstone and Òigh-sgeir pitchstone samples**. See supplementary text for details.*

S4. Detailed contamination history

Relative to the primitive picritic dyke from the Isle of Rum ('M9')[31,84] and the average North Atlantic End Member[48,92,93], which can be viewed as approximations of upper mantle isotope compositions during the Palaeocene (Fig. 7), the Sgùrr of Eigg pitchstone and Òigh-sgeir pitchstone samples show a very strong influence by crustal materials.

The Sr and Nd data are consistent with a model whereby the Sgùrr of Eigg and Òigh-sgeir magmas were derived from a partial melt of the upper mantle that underwent 10-20% contamination by lower crustal granulite-facies-type Lewisian gneiss, followed by up to 50% contamination by upper crustal amphibolite-facies type Lewisian Gneiss (cf. refs. 23, 27, 33, Fig. 7).

Employing the Pb isotope data the quantification of the potential incorporation and contamination of these magmas firstly excludes the Moine (Neoproterozoic) Supergroup as contaminant. As Pb isotopic ratios in the Province generally reflect mixing trends between asthenospheric magmas and lithospheric contaminants (e.g. refs. 48, 82, 93) we note that both the Sgùrr of Eigg pitchstone and Òigh-sgeir pitchstone are much less radiogenic than the radiogenic Moine meta-sedimentary rocks or the assumed mantle end-member. A trend towards Lewisian granulite-facies material is evident, however (Fig. 7a). The OSSEP samples plot where the granulite-facies and amphibolite-facies Lewisian Gneiss fields overlap, remote from the field for the relatively radiogenic Moine rocks and the Palaeogene mantle (Fig. 7). Combined with the identical composition of the Sgùrr of Eigg and Òigh-sgeir pitchstones, the sole contaminants appear to be Lewisian crustal materials, which are restricted to the Hebridean Terrane (Fig. 1). Isotope data in this study thus confirm the initial observation of Dickin and Jones[23] that the Sgùrr of Eigg pitchstone is essentially derived from a large degree of Lewisian crustal input and must be sourced from the Hebridean terrane. Notably, however, the felsic rocks of the Sgùrr of Eigg and Òigh-sgeir pitchstones record a multi-stage evolution, reflecting the cumulative effects of contamination events in both deep and shallow crustal reservoirs (Fig. 7).

S5. Volume estimation of the OSSEP eruptive event

Attempting a first-order estimate for the OSSEP eruptive volume, we can use the direct distances from Skye to Eigg (~41 km) and to Òigh-sgeir (~45 km), together with the pitchstone cross-section on Eigg, like the face exposed at Bidean Boidheach (~150 m in width, ~120 m in thickness). Underlying the Sgùrr of Eigg are conglomerates, giving the valley a U-shape[15,16]. A U-shaped cross-section area was therefore chosen, employing total former valley widths of 300 m (ref. 15), but wider dimensions of 500 m and 750 m have also been considered (Fig. S5; Table S5).

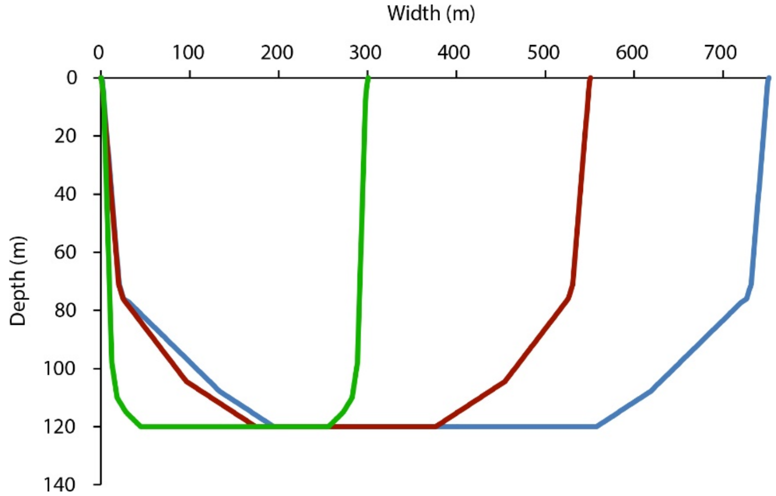

Figure S5: Possible valley shapes and dimensions based on Sgùrr of Eigg field observations. *A 300 m wide (green line), 550 m wide (red line) and a 750 m wide (blue line) valley was used (cf. refs. 15,16) to calculate the volume of a valley fill from Skye to the exposures of the Sgùrr of Eigg and Òigh-sgeir pitchstones (Table S5).*

S6. Possible climate effects of the OSSEP eruption

Employing average magmatic CO_2 values from the literature, the CO_2 potentially released by the OSSEP event calculates to between 0.7 and 0.14 Gt for a range of 5 to 10 km^3 of eruptive volume (cf. ref. 62). This approximates about half the present-day annual volcanic CO_2 emissions (~0.3 Gt, ref. 94). Anthropogenic annual CO_2 emissions in the late 20th century amount to 6.3 Gt/year (ref. 63) and the effects from magmatic CO_2 released by the OSSEP eruption alone may thus have been insufficient to cause large climate effects. Sulfur, on the other hand, may be more important, as it is often enriched in silicic magma relative to basalt[65]. Although we have no compositional data, sulfur contents in melt inclusions in the BPIP are typically between 600 and 800 ppm for primitive basalts[95], comparable to melt inclusions in e.g. Emeishan LIP basalts with sulfur contents of 400 to 1200 ppm[96]. Assuming that the sulfur content in the OSSEP magmas was similar to those of e.g. felsic Emeishan eruptives (≥10,000 km^3 silicic eruptive products released $1x10^{17}$g sulfur into the atmosphere[65]), eruption could have ejected some $1*10^{14}$ g of sulfur into the atmosphere. This amount of sulfur is about an order of magnitude higher than recorded in historical VEI 5 and 6 events, such as Pinatubo 1991 and Krakatau 1883 (estimated sulfur-outputs of $1.5x10^{13}$ and $4.65x10^{13}$ g, respectively, e.g. ref. 25). Interestingly, these two events caused a global temperature decrease of between 0.7 to 1 °C in the years following the eruptions, which implies that the OSSEP eruption could have caused direct climate effects for several years after the event (cf. ref. 25).

Additional references

76. Williamson, I. T. The Petrology and Structure of the Tertiary Volcanic Rocks of West-Central Skye, NW Scotland. (Doctoral dissertation, University of Durham, 1979).
77. Wager, L. R., Vincent, E. A., Brown, G. M. & Bell, J. D. Marscoite and Related Rocks of the Western Red Hills Complex, Isle of Skye. *Philos. Trans. R. Soc. A Math. Phys. Eng. Sci.* **257**, 273–307 (1965). doi: 10.1098/rsta.1965.0006
78. Thompson, R. N. Askja 1875, Skye 56 Ma: Basalt-triggered, Plinian, mixed-magma eruptions during the emplacement of the Western Redhills granites, Isle-of-Skye, Scotland. *Geol. Rundschau* **69**, 245–262 (1980). doi: 10.1007/BF01869036
79. Aboazoum, A. S. A. Petrogenisis of Palaeocene granites, Island of Skye, NW Scotland. (Doctoral dissertation, University of Glasgow, 1995).
80. Thompson, R. N., Esson, J. & Dunham, A. C. Major Element Chemical Variation in the Eocene Lavas of the Isle of Skye, Scotland. *J. Petrol.* **13**, 219–253 (1972). doi: 10.1093/petrology/13.2.219
81. Eiler, J. M. Oxygen Isotope Variations of Basaltic Lavas and Upper Mantle Rocks. *Rev. Mineral. Geochemistry* **43**, 319–364 (2001). doi: 10.2138/gsrmg.43.1.319
82. Dickin, A. P., Jones, N. W., Thirlwall, M. F. & Thompson, R. N. A Ce/Nd isotope study of crustal contamination processes affecting Palaeocene magmas in Skye, Northwest Scotland. *Contrib. to Mineral. Petrol.* **96**, 455–464 (1987). doi: 10.2138/gsrmg.43.1.319
83. Geldmacher, J., Haase, K. M., Devey, C. W. & Garbe-Schönberg, C. D. The petrogenesis of Tertiary cone-sheets in Ardnamurchan, NW Scotland: petrological and geochemical constraints on crustal contamination and partial melting. *Contrib. to Mineral. Petrol.* **131**, 196–209 (1998). doi: 10.1007/s004100050388
84. Upton, B. G. J. *et al.* Picritic magmas and the Rum ultramafic complex, Scotland. *Geol. Mag.* **139**, (2002). doi: 10.1017/S0016756802006684
85. Nicoll, G. R. Evolution and crustal contamination of igneous rocks from the Palaeocene Volcanic District of North-West Scotland. (Unpublished Ph.D. thesis, Trinity College, Dublin, 2008).
86. Ellam, R. M. & Stuart, F. M. The Sub-lithospheric source of North Atlantic Basalts: Evidence for, and significance of a common end-member. *J. Petrol.* **41**, 919–932 (2000). doi: 10.1093/petrology/41.7.919
87. Carmichael, I. S. E. The crystallization of feldspar in volcanic acid liquids. *Q. J. Geol. Soc.* **119**, 95–130 (1963). doi: 10.1144/gsjgs.119.1.0095

88. Meighan, I. G., Gibson, D. & Hood, D. N. Some aspects of Tertiary acid magmatism in NE Ireland. *Mineral. Mag.* **48**, 351–363 (1984). doi: 10.1180/minmag.1984.048.348.05
89. Meighan, I. G., Fallick, A. E. & McCormick, A. G. Anorogenic granite magma genesis: new isotopic data for the southern sector of the British Tertiary Igneous Province. *Trans. R. Soc. Edinb. Earth Sci.* **83**, 227–233 (1992). doi: 10.1017/S0263593300007914
90. Gamble, J. A., Wysoczanski, R. J. & Meighan, I. G. Constraints on the age of the British Tertiary Volcanic Province from ion microprobe U-Pb (SHRIMP) ages for acid igneous rocks from NE Ireland. *J. Geol. Soc. London.* **156**, 291–299 (1999). doi: 10.1144/gsjgs.156.2.0291
91. Macdonald, R., Bagiński, B., Dzierżanowski, P. & Jokubauskas, P. Apatite-supergroup minerals in UK Palaeogene granites: composition and relationship to host-rock composition. *Eur. J. Mineral.* **25**, 461–471 (2013). doi: 10.1127/0935-1221/2013/0025-2291
92. Thirlwall, M. F. & Jones, N. W. Isotope geochemistry and contamination mechanics of Tertiary lavas from Skye, Northwest Scotland. in *Continental Basalts and Mantle Xenoliths* (eds. Hawksworth, C. J. & Norry, M. J.) 186–208 (Shiva, 1983).
93. Dickin, A. P. & Durant, G. P. The Blackstones Bank igneous complex: geochemistry and crustal context of a submerged Tertiary igneous centre in the Scottish Hebrides. *Geol. Mag.* **139**, 199–207 (2002). doi: 10.1017/S0016756802006283
94. Mörner, N.-A. & Etiope, G. Carbon degassing from the lithosphere. *Glob. Planet. Change* **33**, 185–203 (2002). doi: 10.1016/S0921-8181(02)00070-X
95. Peate, D. W., Peate, I. U., Rowe, M. C., Thompson, J. M. & Kerr, A. C. Petrogenesis of High-MgO Lavas of the Lower Mull Plateau Group, Scotland: Insights from melt inclusions. *J. Petrol.* **53**, 1867–1886 (2012). doi: 10.1093/petrology/egs036
96. Zhang, Y., Ren, Z.-Y. & Xu, Y.-G. Sulfur in olivine-hosted melt inclusions from the Emeishan picrites: Implications for S degassing and its impact on environment. *J. Geophys. Res. Solid Earth* **118**, 4063–4070 (2013). doi: 10.1002/jgrb.50324
